# Microfluidic Perfusable Pathological Vasculature for Atherosclerosis Drug Screening

**DOI:** 10.34133/research.0902

**Published:** 2025-09-19

**Authors:** Jing Liu, Mulan Zhu, Na Bai, Nan Huang, Wentai Zhang, Zhilu Yang, Ying Wang

**Affiliations:** ^1^ The Tenth Affiliated Hospital, Southern Medical University (Dongguan People’s Hospital), Dongguan, Guangdong 523059, China.; ^2^ Shenzhen Clinical Medical School, Southern Medical University, Guangzhou, Guangdong 510515, China.; ^3^ GuangZhou Nanchuang Mount Everest Company for Medical Science and Technology, Guangzhou 510670, China.

## Abstract

Current in vitro atherosclerosis (AS) models struggle to stimulate blood flow, limiting their ability to replicate endothelial injury and drug transport in AS development. To address this, we developed a 3-dimensional perfusable atherosclerotic vessel-on-a-chip (3D-PAVoC) platform that mimics vascular structure, blood circulating, and disease microenvironment. The system integrates endothelial cells and smooth muscle cells within a flow-enabled arterial construct, exposed to inflammatory (tumor necrosis factor-α and interleukin-1β) and hyperlipidemic stimuli (oxidized low-density lipoprotein) to recreate AS-prone conditions. Flow-dependent endothelial responses including enhanced cell growth and survival were observed, confirming the importance of hemodynamics in disease modeling. Then, rapamycin (RAP) was used as a model drug to evaluate therapeutic effects in the 3D-PAVoC. Compared to static vessel-on-a-chip models and conventional 2-dimensional cultures, 3D-PAVoC exhibited more pronounced AS pathology and higher RAP half-maximal inhibitory concentration, better reflecting in vivo conditions. The effective RAP dose identified in vitro was validated in apolipoprotein E knockout (ApoE^−/−^) mice, where it partially alleviated AS progression. Transcriptomic analysis revealed RAP-mediated modulation of AS-related gene functions and pathways. Overall, the 3D-PAVoC provides a physiologically relevant platform for anti-AS drug screening, bridging the gap between in vitro testing and in vivo validation, and offering insights into drug action under realistic vascular and pathological conditions.

## Introduction

Cardiovascular diseases (CVDs) pose an important global health threat, causing 10 to 17 million deaths annually, a burden that continues to escalate [1]. Atherosclerosis (AS), driven by chronic inflammation, lipid metabolism disorders, and vascular injury caused by risk factors including hypertension, diabetes, obesity, smoking, and unhealthy diets, serves as the primary pathological foundation of CVDs. These pathological features often lead to severe complications, such as arterial fibrosis, calcification, and plaque formation, posing a serious threat to the patients [2,3]. To date, several therapeutic agents, such as rapamycin (RAP) [4], icariin [1], and colchicine [5], have shown some efficiency in inhibiting the development of AS in clinical practices. However, the underlying mechanisms driving AS progression in its early stages remain poorly understood, highlighting the urgent need for further research to explore effective therapeutic strategies.

Essentially, AS is a chronic vascular disorder critically initiated by elevated low-density lipoprotein (LDL) cholesterol levels and increased oxidative stress due to the disturbances of blood, which leads to the over-retention of oxidized LDL (ox-LDL) as well as the secretion of tumor necrosis factor-alpha (TNF-α) and interleukin-1beta (IL-1β). Under healthy physiological conditions, laminar blood flow exerts shear stress on endothelial cells (ECs), promoting their alignment and maintaining vascular homeostasis by suppressing inflammation and oxidative stress. However, disturbed blood flow, often occurring at arterial bifurcations or curves, generates areas of low and oscillatory shear stress, creating a pro-atherogenic environment. In this case, the disturbed blood flow and excessive infiltration of immune factors into the vascular wall triggers endothelial inflammation and dysfunction, disrupting the barrier function of ECs and thus enhancing vascular permeability, thus accelerating lipid deposition and intima damage [6,7]. Meanwhile, this process drives smooth muscle cells (SMCs) to transition from a contractile to a synthetic phenotype, characterized by enhanced proliferation, migration to the intimal layer, and the secretion of extracellular matrix (ECM) components to form the fibrous cap [8,9]. Thus, the uncontrollable SMC activity contributes to fibrosis, calcification, and the formation of atherosclerotic plaques. Accordingly, the pathogenesis of AS is primarily driven by disturbed hemodynamics of blood, inflammatory mediators, lipid accumulation, and intricate cellular signaling between ECs and SMCs, which are considered several pivotal components for effectively modeling AS disease in vitro. In such cases, traditional approaches to construct AS disease models in vitro, such as 2-dimensional (2D) plate model, lack the 3-dimensional (3D) microenvironment and fluid shear stress, leading to nonphysiological cell behaviors. Animal models provide insights into systemic disease processes but face challenges such as species differences and experimental complexity, limiting human applicability [10]. Consequently, building a dynamic and preferable biomimetic 3D AS model that incorporates specific pathological features in vitro is essential for studying the cellular functions and drug development for AS therapy. Among various emerging strategies, microfluidic-based vascular models offer distinct advantages over conventional 3D printing-based systems for drug screening in AS [11]. While 3D-printed scaffolds allow for customizable architectures, they often lack perfusable channels and fine control over fluid dynamics, features essential for replicating shear-dependent endothelial behavior and hemodynamic cues critical to AS progression. In contrast, microfluidic platforms provide precise regulation of flow conditions, real-time imaging capabilities, and compatibility with low-volume, high-throughput screening [12]. These features enable more physiologically relevant modeling of vascular inflammation, lipid accumulation, and therapeutic responses under pathologically relevant flow environments. Consequently, microfluidics presents a more dynamic and versatile approach for both mechanistic studies and drug evaluation in AS [13].

In recent years, organ-on-a-chip technology, which integrates microfluidics with tissue engineering, has emerged as a prevailing platform for disease research and drug screening, owing to its high biomimicry and precise dynamic control capabilities [14–17]. Among these, vessel-on-a-chip (VoC) has shown great potential in dynamically reconstructing the complex interactions between ECs and SMCs [18,19]. Additionally, integrating hydrogels into VoC systems is considered an effective strategy for creating 3D well-defined architectures with the spatial organization of multiple cell types. For instance, on the one hand, incorporating hydrogels, such as gelatin methacrylate (GelMA), enhances biomimicry and functionality by providing a 3D structure that promotes cell adhesion, growth, and viability [20,21]. On the other hand, its porous, high-water-content network efficiently retains water-soluble factors, such as ox-LDL and inflammatory cytokines, showing significant potential in stimulating pathogenic processes of AS, including inflammation and lipid deposition [22,23]. In our previous work, we demonstrated the construction of the 3D biomimetic AS model based on GelMA hydrogels comprising ECs and SMCs to form a multilayer vascular structure [24]. However, the absence of blood flow stimulation in the model leads to a lack of biomimicry to some extent, particularly regarding the critical role of blood flow in endothelial injury and the transport of drugs during the drug screening process. Indeed, it is still challenging for VoC technology to fully replicate in vivo conditions, especially when using external injection pumps, due to the long-term instability of the model structure and inconsistent shear stress and flow oscillations caused by leakage. Furthermore, the current setup often restricts the ability to simultaneously study multiple factors, such as effective flow, multiple cell interactions, and inflammatory surroundings, limiting the model’s effectiveness in capturing the complex interactions and the multifactorial nature of AS for drug testing.

**Fig. 1.**
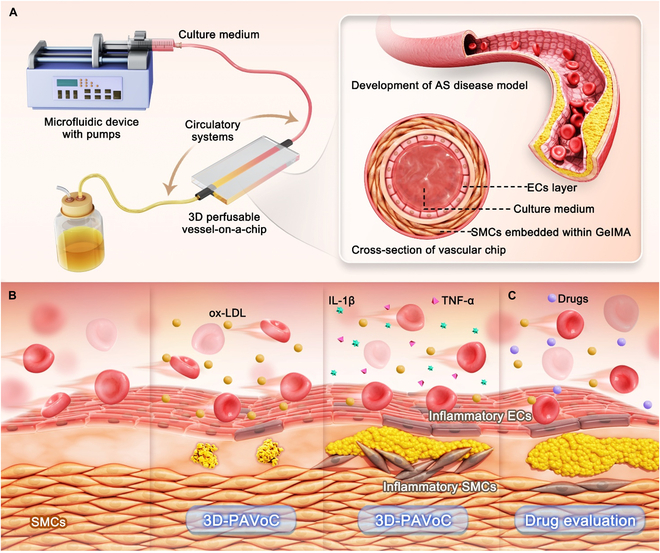
Overview of the study. Schematic illustrating the microfluidic perfusable pathological vasculature for drug screening. (A) The development of a 3D-PVoC system, complete with a circulatory network featuring a vascular structure integrated with ECs and SMCs. (B) The setup for generating a pathological microenvironment for simulating AS conditions. (C) Evaluation of the drug, such as RAP, performance based on the developed 3D-PVoC.

Hence, in this study, we employed the VoC technique and tissue engineering approaches to construct a 3D perfusable atherosclerotic VoC platform (3D-PAVoC) with continuous blood flow through an engineered arterial structure composed of ECs and SMCs. This platform incorporates a highly biomimetic diseased microenvironment with inflammatory stimuli and hyperlipidemic cues, replicating key features of AS-prone conditions for drug evaluation (Fig. 1). As depicted in Fig. 1A, we first developed a 3D perfusable microfluidic vessel-on-a-chip (3D-PVoC) with an external pump that circulates media through the engineered vasculature, composed of multilayered intima and tunica media, arranged from the innermost EC layer to the outermost SMC within a GelMA hydrogel matrix. Subsequently, the development and pathological features of 3D-PAVoC was explored by simulating inflammatory factor induction and lipid accumulation (Fig. 1B). Then, we combined half-maximal inhibitory concentration values against RAP, proteomics, and transcriptomics to analyze the effects of RAP on early AS, alongside drug evaluation using 3D-PVoC without pathological surroundings and traditional 2D monolayer models (Fig. 1C). Through Gene Ontology (GO) functional analysis and the Kyoto Encyclopedia of Genes and Genomes (KEGG) pathway comparison, we identified key pathways and related gene targets, providing new insights into the pathogenesis of AS. To further validate the in vitro findings and establish the translational relevance of the constructed 3D-PAVoC platform, the effective concentration of RAP identified in the system was administered to apolipoprotein E knockout (ApoE^−/−^) mice, a well-established model of AS. This study underscores the platform’s value for drug efficacy prediction, dose optimization, and mechanistic exploration, positioning it as a powerful tool for early-stage drug development in CVD.

## Results and Discussion

### Architectured 3D-PVoC with fluid flow enhancing the biological performance of ECs and SMCs

**Fig. 2.**
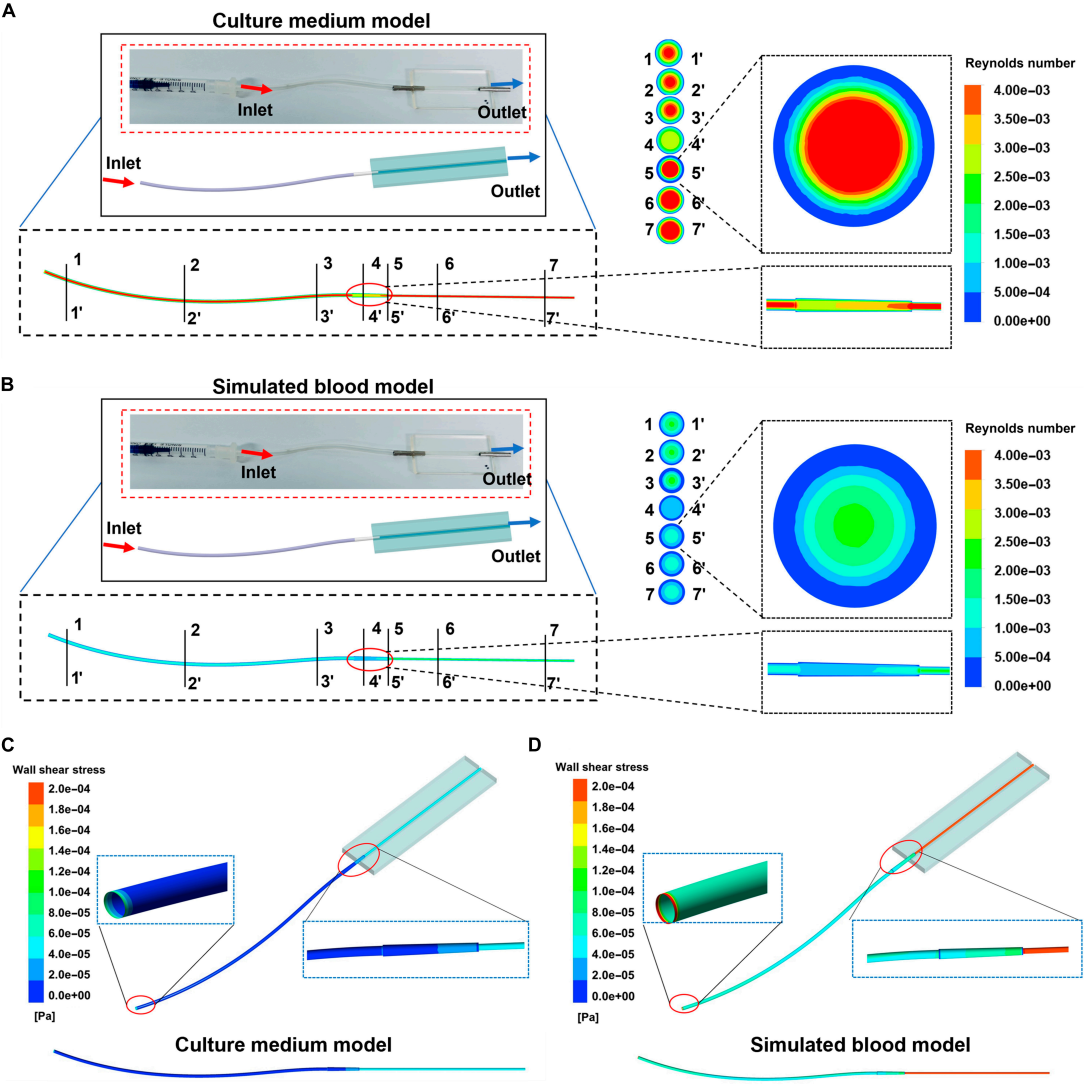
Stimulation of fluid dynamics in the 3D-PVoC. (A) Stimulation of Reynolds number distribution in 3D-PVoC for the culture medium infusion model and (B) the blood infusion model. (C) The wall shear stresses (WSS) distribution in the 3D-PVoC for the simulated culture medium infusion model and (D) the stimulated blood infusion model.

Stimulating blood flow is critical in establishing vascular models, as the shear stress induced by blood flow plays a pivotal role in driving ECs migration, alignment, proliferation, and the overall vascularization process [25,26]. To achieve the shear stress generated by fluid flow into the model [27–29], we have developed a perfusable microfluidic chip system that can stimulate blood flow by integrating an external pump as shown in Fig. S1, enhancing the physiological functions of the cells in vitro to some extent. The size and the assembly of the microfluidic chips are shown in Fig. S2, supporting information. Considering the critical role of wall shear stress in EC function and vascular health, as well as the significance of the Reynolds number in characterizing flow states, we analyzed their effects within the 3D-PVoC channels. While the low Reynolds numbers typically indicate laminar flow, higher values may result in turbulence, increased shear stress, and flow instability on the vascular wall [30]. The shear stress and Reynolds number were calculated and compared between the culture medium and simulated blood flow using fluid dynamics stimulations (Fig. 2). As the channel diameter varies, the Reynolds number of the fluid within the channel changed correspondingly. As shown in Fig. 2A and B, the Reynolds number in the stimulated medium infusion channel and blood infusion remain relatively low across different nodes, indicating a predominantly laminar flow regime. Specifically, the Reynolds number in the blood infusion group is lower than that in the culture medium group due to the higher viscosity of blood compared to the medium. Similarly, the wall shear stress of the culture medium within the channel is relatively uniform and low, whereas the shear stress in the blood infusion group is even lower (Fig. 2C and D).

**Fig. 3.**
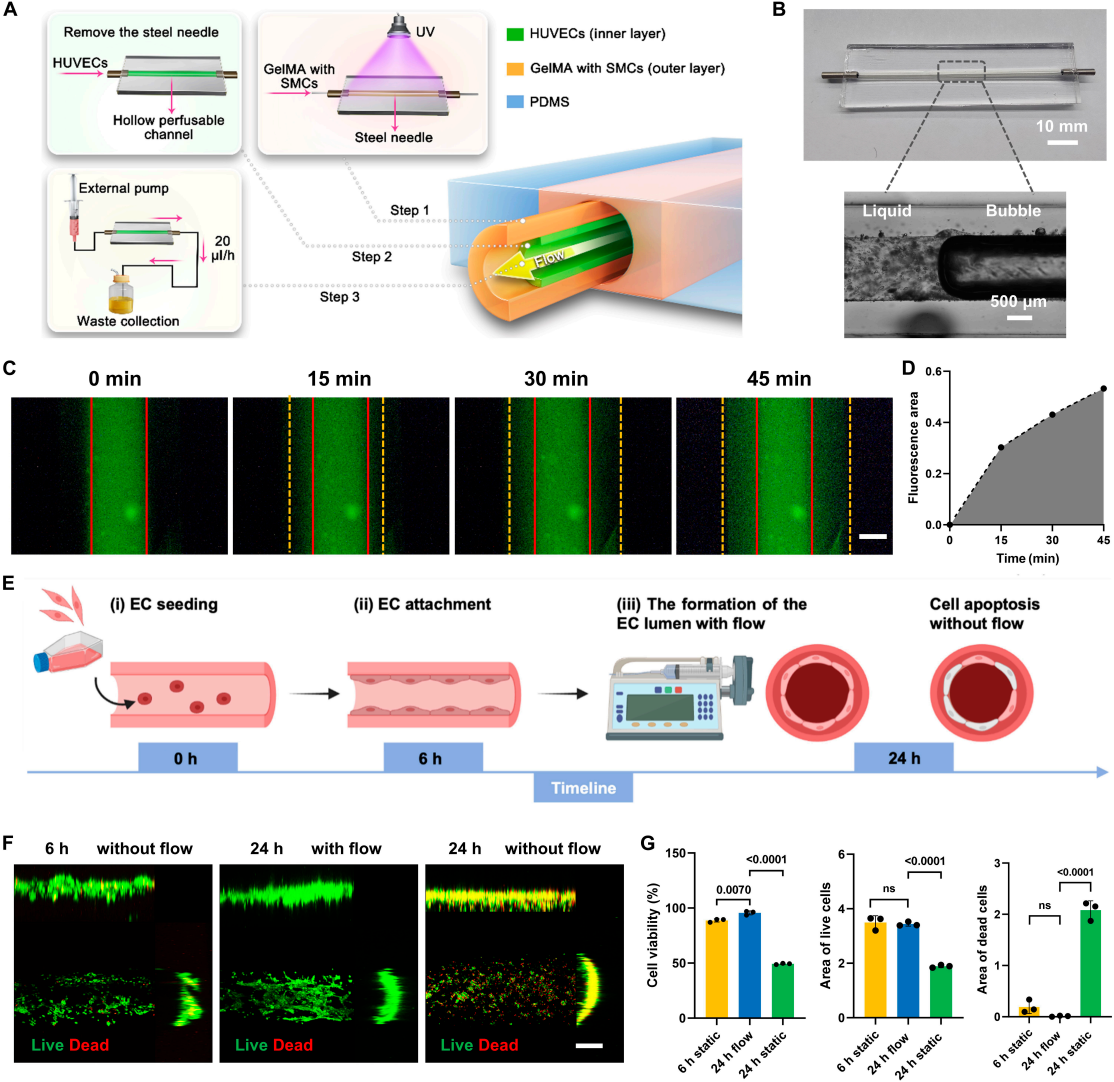
The perfusable media significantly enhancing the cell viability of HUVECs within the hollow architecture, effectively stimulating the vascular lumen structure. (A) Schematic illustration of the 3D-PVoC fabrication steps. (B) Photograph of the PDMS chip alongside electron microscope images illustrating the internal microchannels, with a distinct liquid-bubble interface demonstrating perfusability. (C) Time-lapse fluorescence images showing the diffusion patterns of 10-kDa FITC-dextran from the channel into the surrounding hydrogel bulk. Scale bar, 200 μm. (D) Fluorescence intensity profile of dextran after 45 min of perfusion, with the channel position marked by a solid red line and the extent of dextran diffusion at different time points highlighted by yellow dashed lines. (E) Schematic timeline of EC seeding, attachment, and the formation of EC lumen with the flow of media, along with the group without flow as a comparison. (F) Live/dead staining and (G) quantification of HUVECs in the perfusable channels after 6 h of static culture without flow, 24 h of static culture without flow, and 24 h of fluid perfusion with flow, respectively. Scale bar, 200 μm. Data are presented as mean ± standard deviation (SD). Statistical analysis was performed using one-way ANOVA (*n* = 3).

Essentially, the natural arterial wall comprises 2 main layers, the intima and tunica media, arranged from the innermost to the outmost layer [22]. The innermost layer of the intima consists of a single layer of vascular ECs that play a crucial role in regulating vascular constriction, anticoagulation, and inflammatory responses [31]. The SMCs in the tunica media regulate vessel diameter, provide mechanical support, and maintain vascular tension, thereby ensuring blood flow efficiency and blood pressure stability [32]. Considering the native structure and cellular components of the artery, initially, the tunica media with SMCs were stimulated by injecting GelMA mixed with SMCs into a polydimethylsiloxane (PDMS) chip with an inserted steel needle (Fig. 3). In our previous study, the mechanical properties of the GelMA hydrogel, including Young’s modulus, swelling property, and surface morphology, were thoroughly characterized to evaluate their influence on cell behavior [33]. The Young’s modulus of GelMA increases significantly with higher GelMA concentrations, while the porosity decreases markedly as the concentration increases. Softer hydrogels (low modulus) better mimic the native vascular microenvironment, promoting EC migration, tubulogenesis, and formation of capillary-like networks. The high transparency of PDMS facilitates direct observation of inner structures within the chip (Fig. 3A). However, the intrinsic hydrophobicity of PDMS surface poses challenges for the attachment of cells and water-soluble scaffold materials, such as GelMA. To address this issue, we first performed plasma treatment on the PDMS, followed by adding a poly-L-lysine (PLL) coating for enhanced hydrophilicity [34]. The effectiveness of this modification was evaluated by measuring the water contact angle before and after the treatments (Fig. S3). The results indicated a significant decrease in the contact angle from 112.05° to 92.38° and no significant changes after 48 h, demonstrating an improvement in the hydrophilicity of the PDMS surface after PLL treatment. This enhancement in hydrophilicity increased the adhesion of PDMS with other materials, making it more suitable for scaffold attachment and cell culture applications [34]. Subsequently, the hollow perfusable channel in the center was created by removing the needle, leading to the architecture of the arterial wall. Finally, the human umbilical vein endothelial cells (HUVECs) were introduced and adhered to the surface of the GelMA as well as the middle of the channel to form a ring-shaped intima layer. The PVC tubes connected to the external pumps enabled the perfusion culture of the microfluidic platform. Thus, we have obtained a 3D-PVoC with a structure that stimulates physiological blood vessels in vitro.

Prior to evaluating the biological performance of the cells, the perfusibility of the constructed tube was assessed to confirm the flow of liquid and the injected media. As shown in Fig. 3B, a distinct air–liquid interface was observed in the center of the channel, confirming the presence of an intermediate perfusable channel. The diffusion permeability was then evaluated by analyzing fluorescence intensity at defined positions along the channel axis after 0, 15, 30, and 45 min of injecting fluorescein isothiocyanate (FITC)-labeled dextran solutions. The results shown in Fig. 3C demonstrate that fluorescent dextran progressively diffused from the central channel into the surrounding hydrogel bulk over time, with solid red lines marking the channel interfaces for clarity. In addition, regions near the channel cavity exhibited higher fluorescence intensity, reflecting a greater diffusion rate. The fluorescence intensity profile after 45 min of dextran infusion is depicted in Fig. 3D, further quantifying the extent of diffusion.

To assess cell viability within the perfusable channels, a live/dead assay was conducted on the HUVECs layer after 6 h of static culture to allow for cell attachment, followed by an additional 18 h with media perfusion to support cell proliferation, along with the cells cultured without the perfusion of the media as a comparison (Fig. 3E). The presence of green signals from live cells in Fig. 3F indicates that the HUVECs successfully attached to the surface of the GelMA hydrogel and were generally distributed evenly, which demonstrates the excellent biocompatibility of GelMA hydrogels and suggests their potential to support cell proliferation to some extent. In addition, confocal fluorescence imaging revealed that cells subjected to fluid perfusion after the initial 6-h attachment period and an additional 18-h flow of the media exhibited the highest proportion of viable cells (green), with only a small fraction of dead cells (red), as shown in Fig. 3F. Quantitative analysis of live/dead staining, such as the cell viability and area of the live cells in green, further confirmed significantly higher cell viability in this group, consistent with the fluorescence imaging results. In contrast, the HUVECs in the group without media flow after cell attachment exhibited a larger area of red, indicating dead cells, and yellow, indicating early apoptotic cells, within the channel. This demonstrated a significant reduction in cell viability due to the lack of nutrients and oxygen caused by the absence of fluid media flow, consistent with the significantly increased area of dead cells in Fig. 3G. Together, these results highlighted the critical role of media flow as well as the external pump in maintaining cell viability and proliferation in the constructed hollow architecture, emphasizing the importance of fluid movement in supporting vascular cell survival and function.

**Fig. 4.**
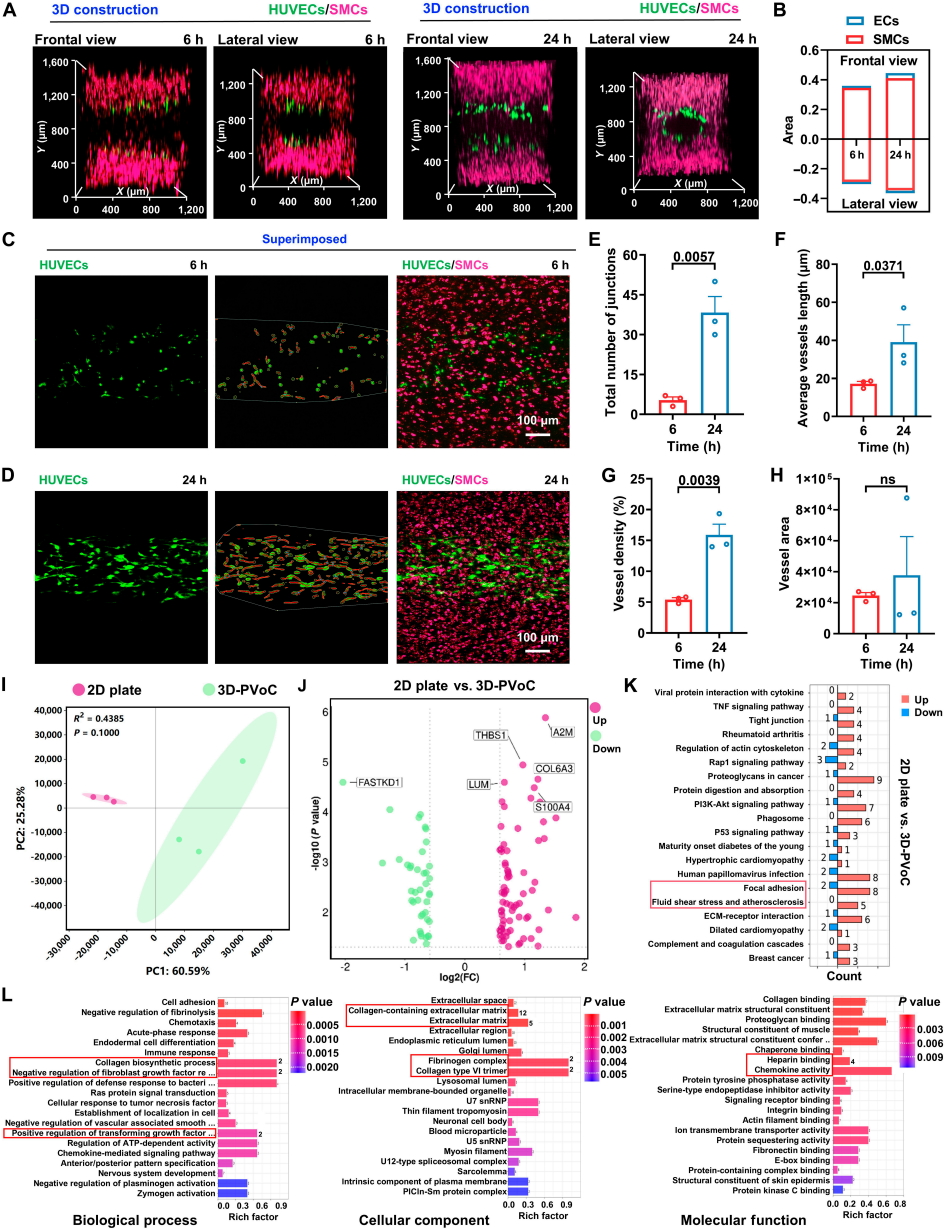
The distribution and biological characterization of GFP-HUVECs and SMCs in the 3D-PVoC. (A) Fluorescence microscopy images showing the distribution of GFP-HUVECs (in green) and SMCs (in purple) in the perfusable VoC, including frontal and lateral views. (B) The fluorescence areas of GFP-HUVECs and SMCs at different time points (6 and 24 h) were measured in the front and side views of the model, respectively. (C and D) CLSM images showing the growth and relative distribution of SMCs and GFP-HUVECs co-cultured in the VoC after 6 h of static culture and 24 h of perfusion culture, respectively. (E to H) Quantitative analysis of angiogenesis of GFP-HUVECs within the VoC was performed using the Angio Tool, and the total number of junctions, average vessel length, vessel density, and vessel area at different incubation times (6 and 24 h) were calculated. Data are presented as mean ± SD (*n* = 3). Statistical comparisons between groups were performed using 2-tailed unpaired Student’s *t* test. “ns” indicates no significant difference. (I) The PCA plot indicating the distribution of samples from the 2D plate and 3D-PVoC. (J) Volcano plots indicating significantly altered proteins in the comparison of the 2D plate and 3D-PVoC. (K) The chart exhibiting the distribution of differentially expressed proteins and genes across various KEGG pathways, highlighting the contrasts between the 2D plate and 3D-PVoC. (L) The most enriched GO terms reveal differences in BP, CC, and MF among the distinct control groups, with all proteins in the pathways highlighted by the red band being up-regulated.

As the constructed perfusable channel demonstrated excellent capability to support cell growth under flow conditions, green fluorescent protein (GFP)-HUVECs (in green) and cell tracker-labeled SMCs (in purple) were utilized to simulate the vascular cellular components for the development of the 3D-PVoC. A confocal laser scanning microscope (CLSM) was initially employed to perform 3D scans of the models to observe the distribution and proliferation of the cells cultured for 6 and 24 h, respectively (Fig. 4). SMCs were observed to be evenly distributed within the GelMA, while GFP-HUVECs exhibited a ring-like distribution at the center of the channel. Both cell types remained spatially separated after 6 and 24 h of perfusion culture using an external pump (Fig. 4A), highlighting the system’s potential to mimic the innermost intimal layer, composed of a single layer of vascular HUVECs, and the SMCs in the tunica media. In addition, the fluorescence areas of both SMCs and GFP-HUVECs showed an increasing trend (Fig. 4B), indicating that GelMA provided a supportive environment for SMC growth and EC attachment. Specifically, the perfusable medium enhanced cell survival and proliferation, enabling effective replication of the vascular structure [35]. Subsequently, Z-stack maximum intensity fluorescence images of HUVECs and SMCs in the 3D-PVoC were merged to visualize cellular connections and potential EC angiogenesis. As shown in Fig. 4C, GFP-HUVECs were sparsely distributed within the model after 6 h in the static culture group, indicating EC attachment to the GelMA surface, consistent with the results in Fig. 2. Additionally, the density of green fluorescence, representing HUVECs, increased significantly, accompanied by a marked enhancement in filopodial connections that extended to establish contact with surrounding cells after further perfusion of the medium (Fig. 4D). The observed cell–cell interactions and the ring-like distribution of GFP-HUVECs demonstrated their self-organizing capability, enabling them to autonomously select optimal growth sites and orientations under media flow at a rate of 20 μl/h [36]. To further quantify these observations and evaluate the impact of the static and flow of the media on angiogenesis, we conducted a detailed angiogenesis data analysis using the Angio tool (Fig. 4E to H). The results showed a significant increase in the total number of junctions, average vessel length, vessel density, and vessel area, with increases of 7.2-fold, 2.3-fold, 2.9-fold, and 1.5-fold, respectively, in HUVECs exposed to media flow for 24 h, compared to cells after 6-h static attachment. These quantifications suggest that the perfusion culture provided essential mechanical stimulation (fluid shear stress), which facilitates EC migration, proliferation, and lumen formation, thereby accelerating the development of vascular networks [26,37,38].

Besides that, the functional protein expression of GFP-HUVECs and SMCs was assessed through fluorescent imaging to confirm the cellular activities and functions within the engineered 3D perfusable biomimetic artery. It was shown that SMCs tracked in yellow gradually extended into a fibrous morphology after 24 h of perfusion culture in GelMA (Fig. S4A), exhibiting cell alignment to some extent, which can be attributed to the properties of GelMA including optimal mechanical characteristics and capacity for matrix remodeling [39]. Meanwhile, the VE-Cad expression of GFP-HUVECs (in purple) exhibited a highly organized ring-like network distribution (Fig. S4B), indicating the formation of stable and orderly connections between GFP-HUVECs that are consistent with the GFP signals. These observations are likely attributed to fluid shear stress, which promotes EC migration and connectivity, thereby enhancing intercellular interactions [37]. Together, the results demonstrate that the engineered hollow structure effectively mimics the natural vascular architecture in vivo. Notably, the perfusable channel with media flow created more physiologically relevant conditions for EC vascularization and SMC proliferation, offering an optimal 3D environment that supports cellular growth and function [40].

To further explore the biological functional changes of the cells occurring in the 3D-PVoC as well as the dynamic perfusable culture approach, we conducted a proteomic analysis comparing the 3D-PVoC with the conventional 2D culture approach. Notably, the 2D plate was cultured under static conditions without AS stimuli, while the 3D-PVoC model was perfused (with flow) but not exposed to ox-LDL, TNF-α, or IL-1β. Both models were maintained under identical basal medium and cell source conditions to ensure comparability. Initially, principal component analysis (PCA) of the proteomics data revealed minimal intragroup variability, suggesting that the samples within each group were highly consistent. However, there were significant intergroup differences, indicating a clear separation between the 3D-PVoC and 2D plate cultures, which highlights distinct proteomic profiles between the 2 culture systems, reflecting the biological and functional changes induced by the 3D environment compared to the traditional 2D model (Fig. 4I). Then, we identified genes that exhibited significantly altered expression levels between the 2D plate and the 3D-PVoC (Fig. 4J). Notably, in the 3D-PVoC, genes such as alpha-2-macroglobulin (A2M), thrombospondin-1 (THBS1), lumican (LUM), and collagen type VI alpha 3 chain (COL6A3) were significantly up-regulated compared to the cells within the 2D plate model. This up-regulation is strongly associated with key biological processes in the group of 3D-PVoC, including ECM remodeling, oxidative stress, and cell proliferation, which plays a crucial role in shaping the structural and functional properties of blood vessels, influencing their development, stability, and response to mechanical forces [41–43]. In this case, the up-regulation genes further validate the feasibility and effectiveness of the vascular model developed in the 3D-PVoC. Subsequently, we performed KEGG pathway analysis to examine the biological pathways enriched in the cells from the 3D-PVoC and 2D plate model as a comparison (Fig. 4K). The focal adhesion pathway showed up-regulation of 8 proteins, indicating that this pathway is significantly activated in the 3D cultured approach. This suggests that cell adhesion-related processes are more actively engaged in the 3D model, likely contributing to enhanced cell–matrix interactions, structural integrity, and cellular behavior within the 3D-PVoC [44]. The activation of focal adhesion signaling in this context may play a crucial role in processes such as migration, proliferation, and tissue organization, which are essential for the formation of functional vascular networks. In addition, 5 proteins were up-regulated in the fluid shear stress and AS pathway, suggesting that the 3D microenvironment more effectively simulates the effects of fluid shear stress, a feature challenging to reproduce in the 2D plate [29]. Furthermore, Fig. 4L presented the GO functional enrichment analysis including biological process (BP), cellular component (CC), and molecular function (MF) and the comparison between 3D-PVoC and the 2D plate model. The 3D-PVoC shows greater enrichment (MF) in GO terms associated with vascular formation, ECM remodeling, inflammatory responses, and intercellular signaling, all of which are closely linked to tissue biomimicry. This highlights the 3D-PVoC’s significant advantage in replicating the in vivo microenvironment and achieving biomimetic functionality. In contrast, the 2D plate primarily exhibits functional enrichment related to basic cellular activities, revealing its limitations in mimicking the complexity of in vivo tissue behavior [45]. Overall, the high biomimicry of the 3D model provides a more reliable in vitro platform for studying vascular development, tissue engineering, and disease modeling.

### The established 3D-PAVoC achieving higher fidelity to pathological features

Our previous work has demonstrated that ox-LDL (50 μg/ml), TNF-α (2 ng/ml), and IL-1β (2 ng/ml) significantly contribute to the development of early-stage AS as immune stimulatory factors [6]. As reported, ox-LDL, as the primary pathogenic factor in atherosclerotic lesions, promotes the progression of AS by inducing EC damage, stimulating the proliferation and migration of SMCs, and triggering inflammatory responses [46]. The pro-inflammatory factors, such as TNF-α and IL-1β, can induce EC apoptosis by activating specific signaling pathways, and significantly promote reactive oxygen species (ROS) production through the regulation of nicotinamide adenine dinucleotide phosphate (NADPH) oxidase and mitochondrial dysfunction [6]. Moreover, these cytokines drive SMCs to transition from a contractile phenotype to a synthetic phenotype, accompanied by an increase in ECM secretion [9]. In this case, to investigate functional changes in HUVECs and SMCs within the 3D-PAVoC, we initially examined the cell-specific VE-Cad for HUVECs. VE-Cad is an adhesion molecule expressed in HUVECs, primarily responsible for maintaining endothelial barrier integrity and regulating cell–cell adhesion strength [47]. To assess VE-Cad expression levels following the application and circulation of pathological factors, we conducted immunofluorescence staining on the 3D-PAVoC, along with the 3D-PVoC model as comparison. Cell area and mean fluorescence intensity were quantified accordingly, as illustrated in Fig. S5. Following treatment with inflammatory factors, a significant reduction in both cell area and fluorescence intensity of VE-Cad was observed in the 3D-PAVoC model, which suggested that inflammatory factors accelerate the progression of AS by down-regulating VE-Cad expression, thereby weakening cell–cell adhesion and disrupting endothelial barrier function to some extent [2]. Furthermore, THP-1-derived M0 MCs were pretreated with ox-LDL (50 μg/ml), TNF-α (2 ng/ml), and IL-1β (2 ng/ml) for 24 h to observe their phenotypic transformation under an inflammatory microenvironment (Fig. S6). As shown in Fig. S6A, untreated cells were generally small and round, whereas M0 MCs exposed to inflammatory stimuli became more flattened, with significant decreased cellular circularity and increased aspect ratio (Fig. S6B). The cells displayed typical “dendritic” or “pseudopod”-like structures characteristic of M1 macrophages (MCs), indicating that M0 MCs exhibited pronounced morphological alterations [48]. These morphological changes support the successful induction of macrophage activation and offer a morphological basis for stimulating MC transformation and inflammatory participation within the 3D-PAVoC system.

**Fig. 5.**
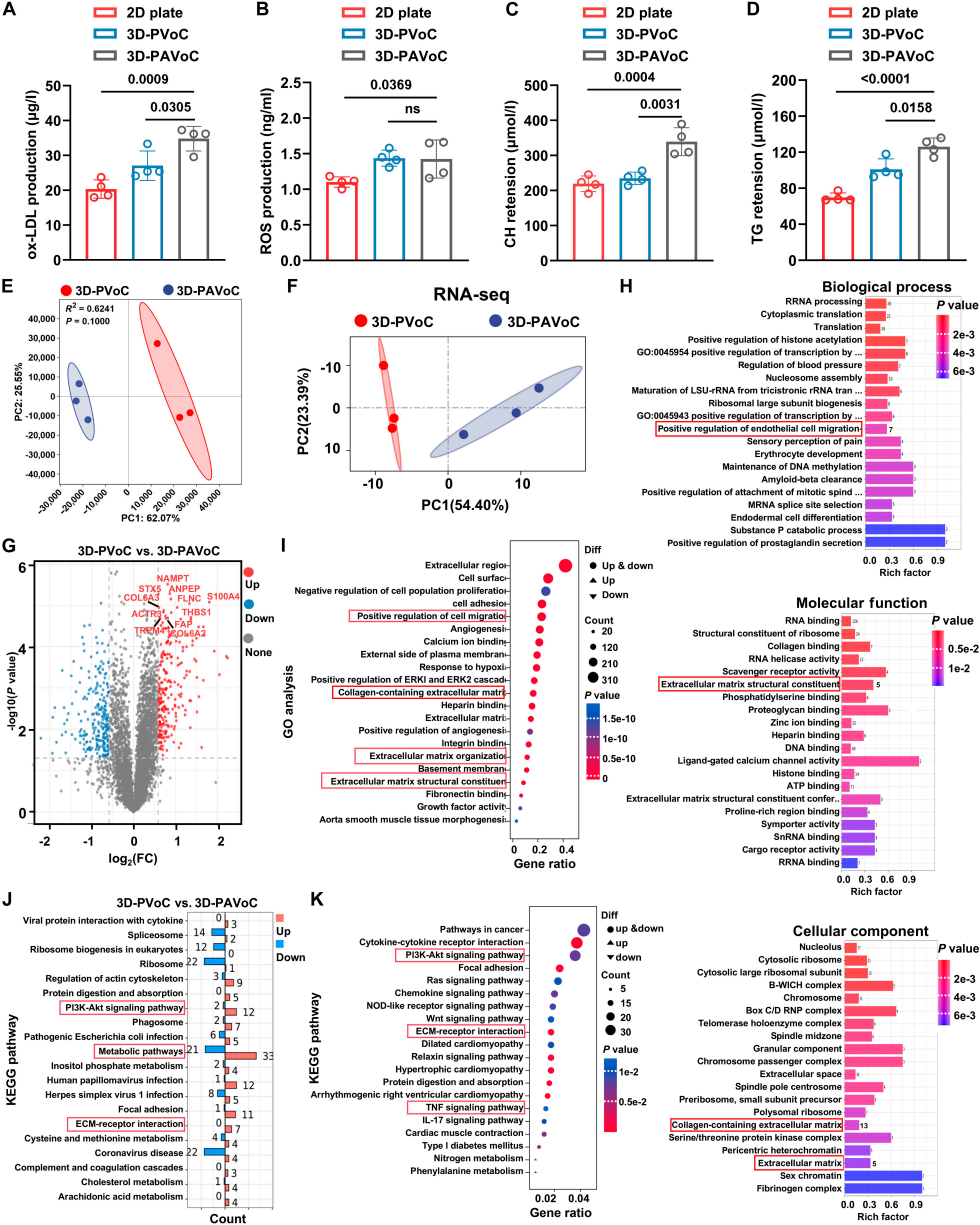
Endothelial dysfunction and bioefficacy evaluation of the 3D-PAVoC. (A to D) Levels of ox-LDL, ROS, TG, and CH in different model groups were determined by ELISA. Data are presented as mean ± SD (*n* = 4). For comparisons between 2 groups, an unpaired Student’s *t* test was used, while one-way ANOVA was applied for comparisons among 3 or more groups. Statistical significance was determined based on *P* values: *P* < 0.05 was considered statistically significant; “ns” indicates no significant difference. (E and F) The PCA plot indicating the distribution of samples from different groups. (G) The volcano plot illustrating proteins with significant differences between the 3D-PVoC and 3D-PAVoC, in which the gray dots represent proteins with nonsignificant differences, and red and blue dots revealing the up-regulated and down-regulated proteins, respectively. (H) The bar chart displaying the GO enrichment analysis results highlights the differences between the control groups across BP, CC, and MF, among which proteins in pathways marked by red boxes are all up-regulated, and (I) demonstrating processes that significantly correlate with changes in gene expression. (J and K) The chart exhibiting the distribution of differentially expressed proteins and genes across various KEGG pathways, highlighting the contrasts between the different experimental groups. The red band highlights pathways associated with the development and progression of atherosclerotic lesions.

Besides that, TNF-α and IL-1β are important pro-inflammatory cytokines that activate HUVECs, promote inflammatory responses, and increase the production of ROS, triggering oxidative stress and leading to EC damage, subsequently boosting the formation of ox-LDL [49]. In turn, as a key factor in the pathological development of AS, ox-LDL is closely associated with plaque formation and elevated levels of total cholesterol (CH) and triglycerides (TG), whose abnormal accumulation accelerates lipid deposition in the vessel wall, driving the progression of AS [50]. Furthermore, CH crystals can activate the NLRP3 inflammasome, leading to the release of inflammatory cytokines such as IL-1β, exacerbating local inflammation and promoting AS progression [51]. Excessive reactive oxygen species (ROS) can lead to oxidative damage and dysfunction of cells, further accelerating the progression of AS [52]. In this case, to validate the bioactivity of the constructed 3D-PAVoC perfused with ox-LDL, TNF-α, and IL-1β, ox-LDL, CH, TG, and ROS levels were measured using enzyme-linked immunosorbent assay (ELISA), along with the 3D-PVoC perfused with fresh media and the co-culture of HUVECs and SMCs in the 2D plate as control groups (Fig. 5). As shown in Fig. 5A and B, compared to the 2D plate, ox-LDL retention and ROS production were higher in the group of the 3D-PAVoC, which indicated significant differences with the *P* values of 0.0009 and 0.0369, respectively, primarily due to the 3D microenvironment being closer to actual in vivo conditions, where the introduction of pro-inflammatory factors enhances oxidative stress, leading to more LDL being oxidized into ox-LDL [2]. Additionally, the increased metabolic activity in the 3D structure and the complex interactions between cells and the ECM further promote ROS generation and LDL oxidation [35]. Meanwhile, CH significantly increased in the 3D-PAVoC (*P* = 0.0031) compared to 3D-PVoC, indicating excess CH deposition in the biomimetic VoC, which might accelerate the probability of formation of the core part of the atheromatous plaque (Fig. 5C) [2]. Additionally, in the 3D-PAVoC, the CVD risk factor, TG, significantly increased after 24 h of inflammatory factor stimulation (*P* = 0.0158), suggesting the promotion of AS to some extent (Fig. 5D) [2].

Moreover, we conducted a comprehensive analysis of the 3D-PAVoC, systematically comparing it to the 3D-PVoC, which lacks a pathological environment, as a control to investigate the transcriptomic and proteomic activities of cells under inflammatory conditions. By integrating tandem mass tag (TMT)-based proteomics and transcriptomics data, we thoroughly explored the dynamic changes in gene and protein expression across the models. PCA of the proteomics and genes data showed minimal intragroup variability but significant intergroup differences, indicating clear separation among the groups (Fig. 5E and F). Subsequently, we identified genes with significantly different expression levels between the pathological and nonpathological microenvironments in the 3D-PAVoC and 3D-PVoC groups, respectively (Fig. 5G). Compared to the 3D-PVoC perfused with fresh media, the 3D-PAVoC exhibits significant up-regulation of numerous genes that are involved in the regulation of inflammatory responses, including nicotinamide phosphoribosyltransferase, S100 calcium-binding protein A4, and THBS1, revealing the great potentials of 3D-PAVoC for studying inflammation-related processes compared to the 3D-PVoC [53]. In addition, the expression of transient receptor potential cation channel subfamily M member 4 (TRPM4) is elevated in atherosclerotic lesions, where it plays a crucial role in calcium signaling, influencing cell proliferation and inflammatory responses within the plaques [54]. The significant up-regulation of TRPM4 in the 3D-PAVoC model suggests that SMCs may adopt fibroblast-like characteristics under inflammatory conditions. This phenotypic shift would potentially lead to enhanced production of collagen and other ECM components, promoting plaque fibrosis and accelerating the progression of AS [55]. Furthermore, the GO analysis results showed that in the proteomics study, the MF category was significantly enriched for ECM structural constituent, while the CC category was significantly enriched for ECM organization and collagen-containing ECM (Fig. 5H). Similarly, the GO analysis of the transcriptomics also revealed significant enrichment of related functions (Fig. 5I), further highlighting ECM remodeling in the 3D-PAVoC compared with 3D-PVoC without pathological surroundings. These findings align closely with the observed characteristics of ECM accumulation and fibrous cap formation in atherosclerotic plaques, highlighting the relevance of the 3D-PAVoC model in mimicking key features of plaque development and progression [2]. In addition, the significant enrichment of positive regulation of cell migration may suggest the regulation of SMC migration and phenotype transformation, consistent with the transition of SMCs to a synthetic phenotype in AS [8]. We finally performed KEGG pathway analysis on 3D-PAVoC with 3D-PVoC as comparison. The differential protein analysis of the 3D-PAVoC revealed significant variations in proteins involved in metabolic pathways, ECM–receptor interactions, and the phosphatidylinositol 3-kinase-Ak strain transforming (PI3K-Akt) signaling pathway (Fig. 5J). The transcriptome results were similarly focused on these pathways (Fig. 5K). These differences revealed the ability of the 3D-PAVoC, with the addition of inflammatory factors, to closely mimic disease mechanisms and cellular behaviors associated with AS [56]. Overall, these observations further validate the successful construction of the 3D-PAVoC following circulation of pathological factors. Compared to the 2D and nonpathological 3D-PVoC controls, HUVECs in the 3D-PAVoC displayed significantly higher ox-LDL retention and ROS production, reflecting enhanced oxidative stress and closer resemblance to in vivo conditions. Transcriptomic and proteomic analyses further revealed activation of inflammation- and ECM-remodeling-related pathways, confirming the model’s capacity to reproduce the disease-relevant EC phenotypes and microenvironment.

### The engineered 3D-PAVoC with circulating pathological conditions enhancing the drug screening efficacy

**Fig. 6.**
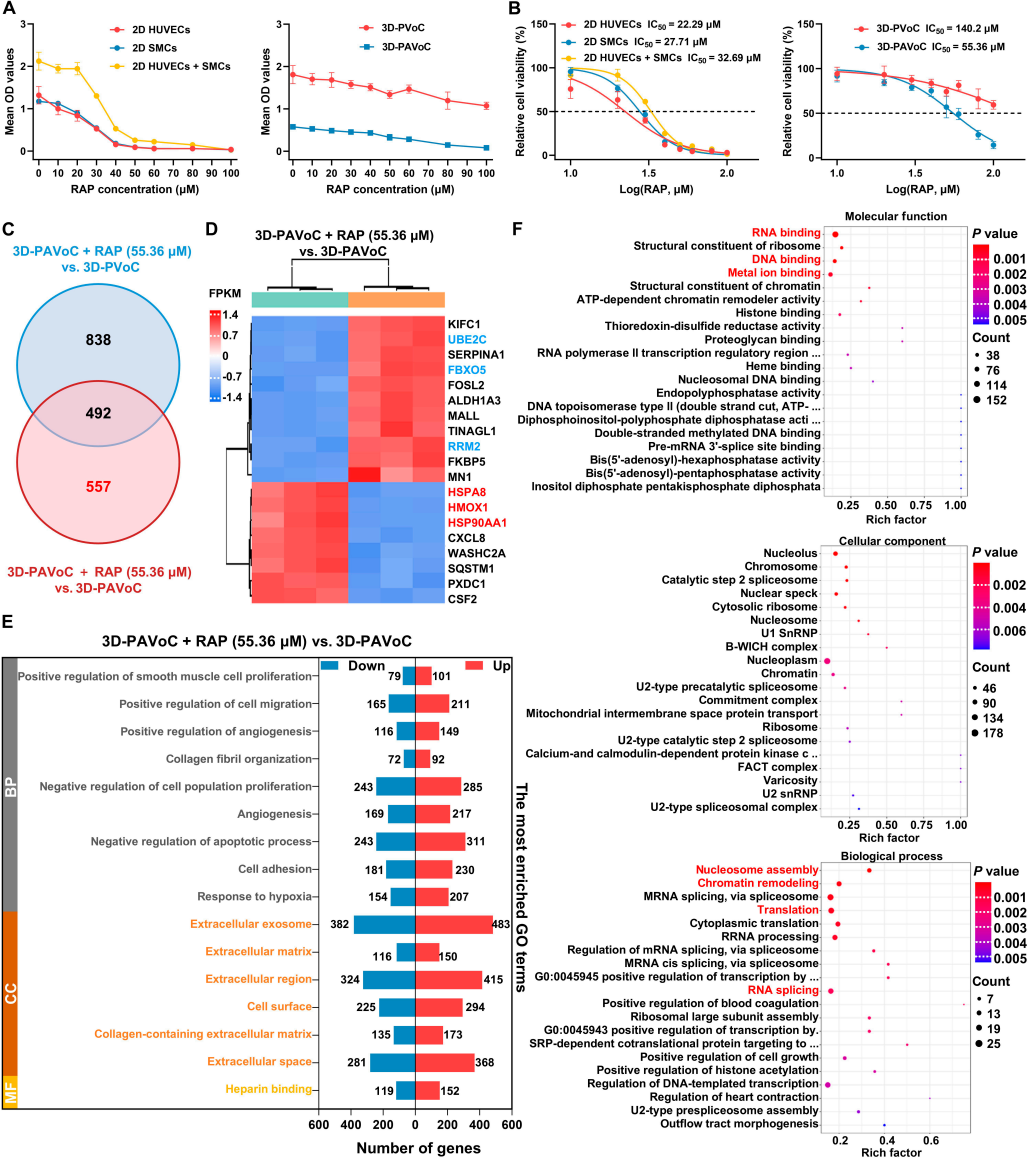
The engineered 3D-PAVoC with circulating pathological conditions enhancing the drug screening efficacy, along with the 3D-PVoC circulating normal media and 2D plate as the comparison. (A) Mean optical density (OD) values after incubation with different concentrations of RAP from 0 to 100 μM. (B) Concentration–response curves and IC_50_ analysis of different concentrations of RAP after incubation in 2D and 3D models, respectively. (C) Venn diagrams illustrate the overlapping DEGs in the 3D-PAVoC with RAP treatment (55.36 μM), compared respectively with the 3D-PVoC and 3D-PAVoC groups. (D) The heatmap displays the top 20 significant DEGs identified from comparisons between 3D-PVAoC with RAP treatment (55.36 μM) and 3D-PVAoC. Blue indicates representative down-regulated genes, while red indicates representative up-regulated genes. (E) GO biological function enrichment analysis of up-regulated and down-regulated genes. GO categories include BP, CC, and MF. (F) GO biological process enrichment analysis of proteins. Red text indicates pathways associated with RAP treatment.

In the early stage of the preclinical drug development process, cytotoxicity approaches, such as cell counting kit-8 (CCK-8) assay, are principally executed to evaluate the safety, efficacy, and pharmacological effects of candidate drugs. In this study, RAP, a drug with unique anti-inflammatory and cell-proliferation regulatory properties, was selected as a model compound to explore its potential therapeutic benefits as predicted by the 3D-PAVoC architecture for AS, alongside drug evaluation using 3D-PVoC and 2D plate models [4]. The CCK-8 assay was used to evaluate cell viability in the 3D-PAVoC model exposed to circulating pathological factors, as well as in the 3D-PVoC model perfused with normal media and the 2D plate as control groups, following RAP treatment at concentrations ranging from 0 to 100 μM. The half-maximal inhibitory concentration (IC_50_) values were determined by fitting the concentration-absorbance data to dose–response curves for each group (Fig. 6A and B). The sensitivities of HUVECs and SMCs to RAP were initially analyzed under a 2D plate, both as single-cell types and in a 1:1 ratio. As depicted in Fig. 6A, the cell viabilities of the different cell types were decreased with the increase in RAP concentration ranging from 0 to 100 μM. Specifically, the cell viabilities of the combined HUVECs and SMCs within this drug concentration range were higher than those of either HUVECs or SMCs alone, indicating a potential protective or synergistic interaction between the 2 cell types in response to RAP treatment. The IC_50_ value for the 1:1 co-culture of HUVECs and SMCs was 32.69 μM, which is higher than that of HUVECs (22.29 μM) and SMCs (27.71 μM) as single-cell types. This result aligns with the findings from the CCK-8 assay. Interestingly, in the engineered 3D models, the IC_50_ values were 140.2 μM for the 3D-PVoC with normal media and 55.36 μM for the 3D-PAVoC with a pathological microenvironment, both of which were higher than the IC_50_ values observed in the 2D plate culture (Fig. 6B). This could be attributed to the enhanced cell–cell and cell–matrix interactions in the 3D models created by GelMA, which better mimic the in vivo environment and may increase cellular resistance to RAP-induced cytotoxicity [39,40]. Notably, the IC_50_ value for the 3D-PVoC with normal media (140.2 μM) is higher than that of the 3D-PAVoC with a pathological microenvironment (55.36 μM), indicating that the pathological conditions make the cells more sensitive to RAP treatment, likely due to increased cellular stress and altered signaling pathways associated with the disease microenvironment [57].

### Altered GO functions and KEGG pathways in response to the drug treatment

To further explore the cellular response to the drug treatment in the group of 3D-PAVoC with a circulating pathological microenvironment, initially, we visualized the distribution of differentially expressed genes (DEGs) between the group of 3D-PAVoC with and without RAP treatment at the concentration of 55.36 μM (Fig. 6C). It was demonstrated that the group of 3D-PAVoC with RAP treatment had 1,330 DEGs compared to the group without RAP treatment, which had 1,049 DEGs, with 492 overlapping DEGs shared between the 2 comparisons, representing the common regulatory effects of the RAP. The heatmap was employed to visualize and display the expression profiles of the top 20 significant DEGs, highlighting several key genes that reveal the potential mechanisms of RAP (Fig. 6D). Among the DEGs, UBE2C, FBXO5, and RRM2, which are involved in the cell cycle and DNA replication, were down-regulated, which suggested that RAP may inhibit cell cycle progression, specifically during the G2/M phase, and reduce pathological proliferation, particularly the abnormal proliferation of SMCs, thereby supporting its anti-atherosclerotic effects [58,59]. The up-regulation of HSPA8, HSP90AA1, and HMOX1 suggested enhanced cellular stress protection, which contributes to mitigate damage to cells within the inflammatory microenvironment [60]. Together, RAP regulated key processes including proliferation, inflammation, and vascular repair, demonstrating its anti-inflammatory and anti-proliferative pharmacological properties while validating the effectiveness of the 3D-PAVoC in simulating pathological features and drug screening.

Then, the most enriched GO terms in Fig. 6E revealed that the 3D-PAVoC with the treatment of RAP at the concentration of 55.36 μM and the ECM-related pathways, including collagen-containing ECM, ECM, and the extracellular region, consist of both up-regulated and down-regulated genes compared with the 3D-PAVoC without drug therapy, which may reflect the dual regulatory effects of RAP on ECM repair or degradation under different conditions. Gene expression differences in inflammation and hypoxia-related pathways, such as response to hypoxia, angiogenesis, and positive regulation of angiogenesis, suggested that RAP may alleviate pathological inflammation and angiogenesis, while moderately enhancing cellular responses to hypoxic stress to protect cells [4]. In pathways associated with the negative regulation of proliferation and migration, such as negative regulation of the apoptotic process, negative regulation of cell population proliferation, positive regulation of cell migration, and positive regulation of SMC proliferation, the down-regulation of certain genes suggested that RAP may inhibit pathological cell proliferation (aberrant SMC proliferation), while up-regulated genes may participate in the repair process or restore normal proliferation [61]. The coexistence of both up-regulated and down-regulated genes might be attributed to differential cellular responses to the RAP, where RAP inhibits SMC proliferation but promotes EC repair, resulting in an overall anti-pathological effect. Further, the proteomics GO analysis results of the group of 3D-PAVoC with the treatment of RAP indicate that drug intervention primarily exerts its effects by regulating post-transcriptional processes (e.g., RNA splicing), epigenetic modifications (e.g., chromatin remodeling), and cellular metabolic balance (e.g., translation and metal ion binding) (Fig. 6F). RAP treatment significantly enhanced activities related to nucleosome assembly, chromatin-associated regions, and RNA/DNA binding functions, demonstrating the potential of drug in restoring gene expression and metabolic balance under pathological conditions by influencing transcription initiation, mRNA processing, and protein expression regulation [62]. Furthermore, the enrichment of metal ion binding implied that the RAP may play a critical role in alleviating oxidative stress and inflammation [63]. Together, these findings provided new insights into the drug’s mechanism of action, highlighting chromatin modifications and RNA-binding proteins as potential therapeutic targets for AS.

**Fig. 7.**
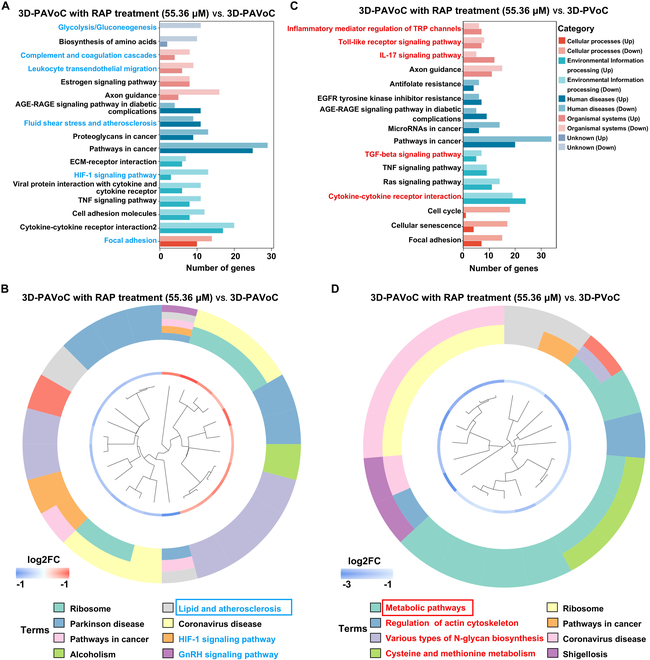
KEGG pathway analysis evaluating the therapeutic effects of RAP (55.36 μM) in the engineered 3D perfusable vascular models. (A and B) KEGG pathway analysis of transcriptomic and proteomic changes in the pathological model with and without RAP treatment: 3D-PAVoC (untreated) vs. 3D-PAVoC with RAP treatment (55.36 μM). (C and D) KEGG pathway analysis comparing the healthy control and the RAP-treated pathological model: 3D-PVoC (untreated) vs. 3D-PAVoC with RAP treatment (55.36 μM). Pathways potentially involved in RAP-mediated regulation of AS progression highlighted in blue and red text, with bar plots enclosed in rectangular boxes to emphasizing specific pathways associated with AS development.

KEGG pathway analysis of the RAP-treated 3D-PAVoC highlighted transcriptomic changes predominantly within 4 major categories: cellular processes, environmental information processing, human diseases, and organismal systems (Fig. 7). The major pathways altered in the transcriptome in the RAP treatment (55.36 μM) group compared with the pathological 3D-PAVoC without treatment included fluid shear stress and AS, HIF-1 signal pathway, focal adhesion, complement and coagulation cascades, leukocyte transendothelial migration, and glycolysis/gluconeogenesis (Fig. 7A). These pathway changes suggested that RAP exerts its therapeutic potential for AS through multiple mechanisms. The genes that up-regulated and down-regulated within the pathway are detailed in Table S1. Among others, shear stress, the force exerted by blood flow on the vessel wall, critically influenced AS progression by driving vascular remodeling and plaque formation [18]. In this case, RAP may mitigate endothelial dysfunction and inflammation induced by shear stress, reducing damage and contributing to the attenuation or reversal of AS development. The focal adhesion pathway mediated cell–ECM interactions, and RAP may suppress SMC proliferation and migration, thereby mitigating pathological vascular remodeling and slowing AS progression [8]. RAP may regulate complement and coagulation cascades to reduce immune activation and thrombosis while promoting vascular repair, addressing key inflammatory and vascular injury mechanisms in AS [3]. Furthermore, RAP may inhibit leukocyte migration, decreasing inflammation in the vessel wall and thereby attenuating plaque formation [6]. Notably, proteomic pathway alterations also focus on lipid metabolism, HIF-1 signaling pathway, and GnRH signaling pathways, further supporting RAP’s potential in treating AS (Fig. 7B). The lipid and AS pathway mainly includes the genes such as CASP6, CYCS, CAMK2D, CDC42, TLR2, HSPA1L, CYBA, R3HDM4, CXCL1, and CAMK2G. RAP may regulate the inflammatory state and metabolic state of cells by inhibiting the mechanistic target of RAP pathway, thereby causing changes in TLR2, CXCL1, HSPA1L, and CYBA [4]. Additionally, the HIF-1 pathway, closely linked to oxidative stress and cellular responses to hypoxia, suggests that RAP may alleviate endothelial damage and vascular remodeling caused by hypoxic conditions [62]. Taken together, RAP demonstrates therapeutic potential in slowing or reversing AS progression through multiple mechanisms targeting key pathways closely associated with AS.

To further investigate the therapeutic effects of RAP, we compared the variations in KEGG pathways between the RAP-treated group and the healthy vascular architecture group, 3D-PVoC. It was shown that the KEGG pathways closely related to the RAP treatment vs. 3D-PVoC include the inflammatory mediator regulation of TRP channels, toll-like receptor signaling pathway, IL-17 signaling pathway, transforming growth factor-beta (TGF-β) signaling pathway, and cytokine–cytokine receptor interaction (Fig. 7C). The inflammatory mediator regulation of transient receptor potential (TRP) channels pathway, which regulates TRP channels by inflammatory mediators, may play a crucial role in RAP treatment by modulating intracellular calcium flow and subsequently influencing cellular inflammatory responses [64]. Additionally, the cytokine–cytokine receptor interaction pathway, a core regulator of immune and inflammatory responses, involved the signaling of various cytokines, such as IL-1 and TNF [3]. The genes that up-regulated and down-regulated within the pathway are detailed in Table S2. RAP may modulate this pathway to exert anti-inflammatory and immune-regulatory effects, thereby helping to suppress pathological inflammation and improve vascular repair [4]. The TGF-β signaling pathway played an essential role in cell proliferation, differentiation, and fibrosis. RAP may modulate the TGF-β pathway to inhibit the proliferation and transformation of SMCs, reducing pathological vascular remodeling and slowing the progression of AS. Furthermore, proteomic KEGG analysis revealed that downstream changes in protein pathways were predominantly associated with the following pathways: metabolic pathways, regulation of the actin cytoskeleton, various types of N-glycan biosynthesis, and cysteine and methionine metabolism (Fig. 7D). These changes complement the transcriptomic findings, where modulation of these protein pathways likely contributes to the anti-inflammatory and immune-regulatory effects of RAP treatment. For example, the regulation of the actin cytoskeleton is crucial for cell movement and structural integrity, which may support vascular repair processes [65]. Meanwhile, metabolic pathways, N-glycan biosynthesis, and cysteine and methionine metabolism collectively supported the therapeutic potential of RAP in maintaining cellular homeostasis, modulating inflammation, and preventing AS progression by regulating energy balance, protein function, and redox detoxification. These protein-level changes were consistent with the transcriptomic alterations observed, linking the molecular mechanisms at both the gene and protein levels to RAP’s therapeutic effects.

### Anti-atherosclerotic efficacy of RAP in vivo

**Fig. 8.**
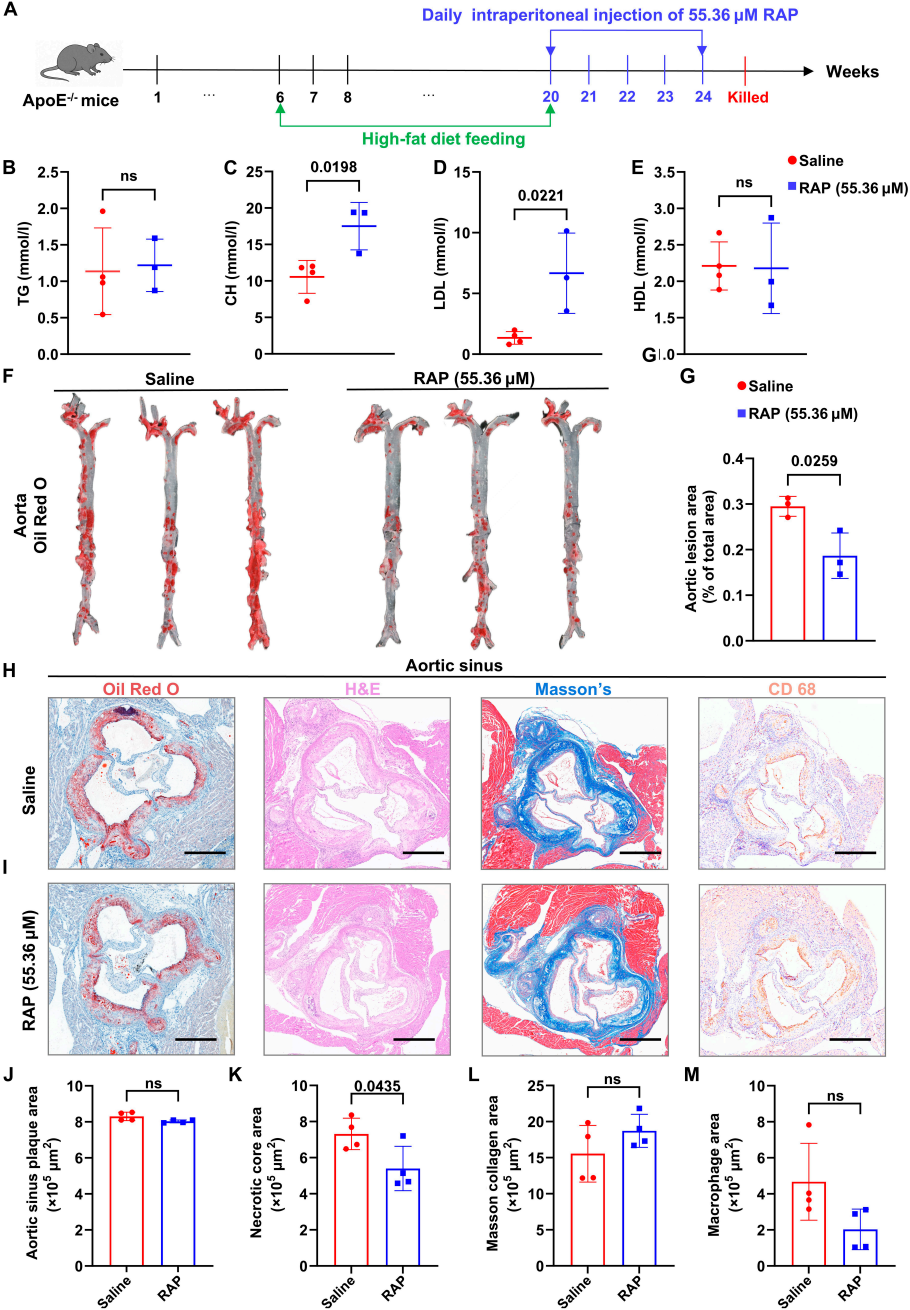
Evaluation of the anti-atherosclerotic effects of RAP in ApoE^−/−^ mice based on lesion quantification, along with the treatment of saline as the comparison. (A) Schematic illustration of the experimental timeline and treatment protocol. (B to E) Plasma lipid profiles including TG, CHO, LDL-cholesterol (LDL-C), and HDL-C following the treatment of RAP (*n* = 4). (F) Representative en face images of aortas stained with ORO following different treatments. (G) Quantification of ORO-positive lesion area as a percentage of the total aortic surface area (*n* = 3). (H and I) Representative histological images of aortic sinus sections stained with ORO, H&E, Masson’s trichrome, and CD68 antibody. Scale bar, 500 μm. (J to M) Quantitative analysis of plaque area (J), total necrotic core area (K), collagen content (L), and macrophage-positive area (M) in atherosclerotic lesions. RAP was employed at the concentration of 55.36 μM. Data are presented as mean ± SD (*n* = 4). Statistical comparisons between groups were performed using 2-tailed unpaired Student’s *t* test. “ns” indicates no significant difference.

Based on the effective concentration of RAP identified using the 3D-PAVoC platform, we further evaluated its therapeutic efficacy in vivo employing a well-established ApoE^−/−^ mice model of AS (Fig. 8A) [66,67]. Following 4 weeks of RAP administration, histological evaluation of major organs, including the liver, kidney, heart, and lungs, revealed no observable tissue damage or signs of systemic toxicity (Fig. S7), indicating that RAP is well tolerated in vivo. To dynamically monitor vascular structural changes in vivo, longitudinal B-mode ultrasound imaging of the aortic root was performed in wild-type and ApoE^−/−^ mice subjected to treatment, either RAP or saline as control. In wild-type mice, the aortic architecture remained stable from weeks 21 to 24, indicating preserved vascular integrity under physiological conditions (Fig. S8A). In contrast, ApoE^−/−^ mice receiving saline displayed progressive aortic wall thickening and luminal narrowing over the 4-week observation period, reflecting the natural course of atherosclerotic lesion development in this model (Fig. S8B). Notably, ApoE^−/−^ mice treated with RAP showed a marked attenuation of structural deterioration, characterized by reduced wall thickening and better-maintained lumen morphology relative to saline controls (Fig. S8C) [17,68]. These results highlight the ability of RAP to preserve vascular structure in vivo and suggest its therapeutic potential in mitigating AS-induced vascular remodeling. Then, the serum lipid profiles were assessed, such as TG, CH, LDL, and high-density lipoprotein cholesterol (HDL-C), to evaluate the systemic metabolic impact of RAP [69–71]. As shown in Fig. 8B to E, compared to the saline group, RAP treatment did not significantly alter TG or HDL-C levels. However, significant increases in CH (*P* = 0.0198) and LDL (*P* = 0.0221) were observed in the RAP-treated mice, suggesting a potential effect of RAP on cholesterol metabolism [72].

In addition, to assess the direct impact of RAP on atherosclerotic lesion development, we performed en face Oil Red O (ORO) staining of the entire aorta **(**Fig. 8F**)**, which enables lipid-specific visualization and quantification of atherosclerotic burden across the vascular surface. It showed that RAP treatment led to a significant decrease in plaque coverage compared to the injection of saline after 4 weeks, indicating its efficacy in attenuating atherosclerotic lesion development and suggesting a direct protective effect on vascular health independent of systemic lipid modulation (Fig. 8G) [73]. To further investigate the plaque composition and structure, we analyzed serial cross-sections of the aortic sinus, a region prone to lesion development in ApoE^−/−^ mice, which were subjected to ORO staining for lipid accumulation, hematoxylin and eosin (H&E) for tissue morphology, Masson’s trichrome staining for collagen deposition, and immunohistochemical staining for cluster of differentiation 68 (CD68), a marker of macrophage infiltration (Fig. 8H and I). ORO staining of the sinus region corroborated the en face findings, showing a marked reduction in lipid-rich lesion areas in RAP-treated mice (Fig. 8J). H&E staining revealed that RAP-treated mice had significantly smaller necrotic core areas within plaques (Fig. 8K). The reduction in necrotic core size indicates that RAP potentially suppresses pathological lesion progression and promotes a more stable plaque phenotype [74]. In addition, Masson’s trichrome staining revealed that RAP treatment significantly enhanced collagen deposition (stained blue), with the highest collagen area observed in this group (Fig. 8L). A robust fibrous cap is essential for preventing plaque rupture and subsequent thrombotic events, suggesting that RAP not only inhibits plaque growth but also reinforces structural integrity [75,76]. Finally, immunohistochemical staining for CD68 was performed to evaluate macrophage infiltration—a key driver of inflammation, matrix degradation, and plaque instability. RAP treatment significantly reduced CD68-positive areas within the plaques (Fig. 8M), indicating effective suppression of macrophage-driven inflammatory responses. Given that MCs orchestrate the inflammatory cascade in AS and contribute to necrotic core formation and fibrous cap thinning, this result further supports the anti-inflammatory and plaque-stabilizing potential of RAP [77].

Together, these comprehensive histological and biochemical analyses demonstrate that RAP treatment significantly reduces lesion burden, suppresses vascular inflammation, enhances collagen-mediated plaque stability, and limits necrotic core formation-without eliciting systemic toxicity. Notably, the therapeutic efficacy observed in vivo was achieved using the optimal RAP concentration at 55.36 μM initially identified through our 3D-PAVoC platform. This underscores the translational potential of our system not only for modeling disease-relevant vascular phenotypes but also for efficiently screening and optimizing drug dosing regimens. These findings position RAP as a promising candidate for the treatment of advanced AS and validate the 3D-PAVoC platform as a valuable tool for preclinical drug discovery and development.

## Discussion

In this study, we successfully developed a 3D-PAVoC model with continuous flow, allowing the circulation of pathological factors to drive AS development and facilitate drug transport. Initially, the development of a PDMS chip with a hollow structure, along with the incorporation of GelMA hydrogel, enabled the formation of a vascular structure stimulating both the intima and tunica media by integrating HUVECs and SMCs, respectively. This setup provides a more physiologically relevant environment compared to traditional 2D plate model. Based on this setup, the circulating lipid factors, such as ox-LDL, and pro-inflammatory cytokines, TNF-α and IL-1β, promoted the pathological features including significant higher expression of ox-LDL, ROS, CH, and TG, along with the 3D-PVoC without AS-prone conditions and 2D cell monolayer as the comparisons. Subsequently, RAP was selected as a model compound to explore its potential therapeutic benefits, showing a higher IC_50_ value in the 3D-PAVoC group (55.36 μM) compared to the 2D plate model (32.69 μM). This discrepancy may not solely reflect biological differences in drug sensitivity but could also be influenced by the physicochemical characteristics of the 3D system. In particular, the GelMA hydrogel matrix used in the 3D-PAVoC platform may act as a diffusion barrier, limiting drug penetration and thereby reducing the effective concentration of RAP reaching the embedded cells.

Then, we incorporated RAP (55.36 μM) into the 3D-PAVoC to evaluate its therapeutic potential. The inclusion of RAP resulted in significant alterations in both the transcriptomic and proteomic profiles, affecting several crucial pathways involved in AS progression. Specifically, RAP was found to modulate pathways such as inflammatory mediator regulation of TRP channels, toll-like receptor signaling, and cytokine–cytokine receptor interaction, which are central to immune activation and vascular inflammation in AS. Additionally, RAP influenced metabolic pathways, including glycolysis/gluconeogenesis and biosynthesis of amino acids, indicating its potential to improve cellular metabolism and reduce metabolic stress in AS-affected vessels. Through this perfusable and integrated platform, we identified several key druggable targets within these pathways, suggesting that RAP could reduce pathological inflammation, inhibit SMC proliferation and migration, and promote endothelial repair, ultimately slowing or potentially reversing AS progression. These findings demonstrate the utility of the 3D-PAVoC in studying AS and provide valuable insights into the molecular mechanisms through which RAP exerts its effects, offering a promising avenue for future therapeutic strategies targeting AS. Moreover, although the 3D-PAVoC system successfully integrates perfusable vascular architecture, inflammatory and lipidic stimuli, and flow-induced shear stress to model pathological AS progression, the study did not include direct comparisons with existing 3D perfusable vascular chip models or other advanced microfluidic-based vascular platforms. This limits the ability to fully demonstrate the platform’s technical innovation and distinct functional advantages. To address this, future studies should incorporate side-by-side benchmarking against state-of-the-art vascular chip systems under standardized conditions, thereby providing a more rigorous validation of the 3D-PAVoC platform’s performance and unique contributions.

To further validate the predictive and translational feasibility of the engineered 3D-PAVoC platform, we applied the IC_50_ concentration of RAP (55.36 μM), as determined in vitro using the 3D-PAVoC system, to the ApoE^−/−^ mouse model of AS to validate the therapeutic effects of the RAP as well as the utility of the 3D-PAVoC model for drug evaluation. Notably, this concentration—screened under dynamic conditions based on the 3D-PAVoC system—partially alleviated disease progression in vivo, as evidenced by reduced plaque burden, smaller necrotic core areas, increased fibrous cap collagen content, and decreased macrophage infiltration. These in vivo outcomes closely mirrored the phenotypic and pathway-level changes observed in the 3D-PAVoC model, further supporting its relevance for preclinical drug evaluation. Despite the strengths of the 3D-PAVoC platform in recapitulating early atherosclerotic microenvironments and enabling physiologically relevant drug screening, certain limitations should be acknowledged. In particular, the IC_50_ value obtained from the 3D-PAVoC model (55.36 μM) was directly used to guide the in vivo RAP dosing in ApoE^−/−^ mice. This approach might partially overlook the complexity of pharmacokinetic processes that influence in vivo drug efficacy, including absorption, distribution, metabolism, and excretion. Although the therapeutic benefit of RAP was successfully validated in vivo, future studies should incorporate comprehensive pharmacokinetic and pharmacodynamic analyses to refine dosing strategies and enhance the clinical translatability of the platform. Moreover, the current 3D-PAVoC model lacks direct perfusion of monocyte-derived MCs, preventing dynamic observation of their adhesion and transendothelial migration. This limitation hampers comprehensive stimulation of immune cell–endothelium interactions that are pivotal in atherosclerotic progression.

Looking ahead, the 3D-PAVoC platform holds significant promise for future applications in cardiovascular research and drug discovery. For instance, its integration into multiorgan chip systems could facilitate the study of interorgan communication and systemic responses, an essential aspect of complex diseases such as metabolic syndrome and systemic inflammation. Although the 3D-PAVoC model captures essential pathophysiological features, it has not yet been directly compared with other emerging in vitro systems, such as AS-related organoids. While organoid models effectively replicate multicellular crosstalk and tissue-level complexity, they typically lack perfusion and mechanical cues that are critical for accurately modeling vascular pathologies. Future comparative or integrative studies between the 3D-PAVoC system and organoid- or chip-based models may reveal their respective strengths and limitations, thereby guiding further innovation in disease modeling and personalized therapeutic screening.

To deepen clinical translation, these findings suggest that the 3D-PAVoC platform could also be further leveraged to stratify patient-specific responses by integrating patient-derived cells or pathological cues, ultimately enabling personalized drug screening. Moreover, the strong concordance between in vitro and in vivo outcomes lays a foundation for incorporating this platform into early-phase preclinical pipelines, potentially reducing the reliance on extensive animal testing and accelerating the identification of effective therapeutic candidates for clinical evaluation.

In summary, we established an in vitro drug screening platform that stimulates the pathological conditions of AS. Unlike traditional 2D or static 3D models, the perfusable and pathologically integrated 3D-PAVoC system more faithfully recapitulates the vascular microenvironment of early-stage AS, enabling more physiologically relevant drug screening. The successful in vivo validation of the RAP concentration identified in vitro highlights the platform’s capacity not only to stimulate complex disease features but also to define clinically meaningful therapeutic windows.

## Materials and Methods

### Materials

GelMA (GM-30), lithium phenyl-2,4,6-trimethylbenzoylphosphinate (LAP), GelMA lyophilized powder (GM-LS-001), and UV light (405 nm, 3 W) were purchased from EFL (Suzhou, China). Poly(methyl methacrylate) (PMMA), PDMS films, polyvinyl chloride (PVC) connecting tubes (1.8 mm inner diameter, 2 mm outer diameter), and adapters were purchased from Wenhao Microfluidic Technology Co., Ltd. (Suzhou, China). HUVECs, GFP-HUVECs, human SMCs, and DAPI were obtained from Guangzhou Tianzhi Biotechnology Co., Ltd. Smooth muscle cell medium (SMCM) and EC medium were purchased from ScienCell (Carlsbad, USA). Penicillin–streptomycin, fetal bovine serum (FBS), F-12, and RPMI 1640 were purchased from Gibco (Grand Island, USA). Dulbecco’s modified Eagle’s medium and phosphate-buffered saline (PBS) were purchased from Vivacell (Denzlingen, Germany).

### The assembly of the perfusable microfluidic system

The chip model, measuring 20 mm in length, was designed with a linear microchannel structure using AutoCAD software (Autodesk, California, USA) and made in PMMA. The dimensions of the microchannel within the chip model are 2 mm (width) × 2 mm (height) × 80 mm (length). To fabricate the perfusable microchannel with the chip model, in brief, the PDMS polymer (Dow Corning, Michigan, USA) was poured into the PMMA chip model then defoamed for 15 min and cured for 1 h (with a temperature decrease from 80 to 25 °C) in a PDMS mixing, defoaming, and curing integrated machine (Wenhao Co., Ltd, WH-HTG-01, China). Once cured, peel the PDMS from the PMMA mold to obtain the bottom layer of the chip. Next, a commercial PDMS film with a thickness of 100 μm was selected as the top layer of the chip. This top layer, along with the bottom layer, was placed into the plasma cleaner (Mycro, PDC-002, USA) for oxygen plasma treatment to bond the layers tightly together. Then, connect 316L stainless steel tubes, with an outer diameter of 2.2 mm, a wall thickness of 0.2 mm, and a length of 15 mm, to both ends of the chip channel, allowing the connection to the PVC tubes as well as 2 external pumps at the ends, thus forming a perfusable culture system. Finally, the connection is sealed with high-transparency UV adhesive (Kisling AG, Wetzikon, Switzerland). Subsequently, one end of the PVC tube was connected to a syringe filled with culture medium via an adapter and placed on an external pump, with a flow rate set to 20 μl/h for the perfusion culture of the chip. The other end was connected to the waste collection. To assess the diffusivity of the perfusable channel, a culture medium containing 25 μg/ml FITC-labeled dextran (Macklin, China) was introduced into the construct. The FITC-dextran was allowed to diffuse from the lumen into the GelMA layer for 45 min. Diffusion patterns were monitored using a Zeiss Axio Observer 3 inverted fluorescence microscope (Germany). Fluorescence images were captured at 0, 15, 30, and 45 min, and the fluorescence intensity within the microfluidic structure was quantified using ImageJ software.

### Fluid dynamics stimulation of culture medium and blood in a 3D-PVoC

We used ANSYS Fluent version 2023 (ANSYS, Lebanon, NH, USA) to compare the fluid dynamics of culture medium and blood in a 3D-PVoC. To isolate the effects of fluid type, all simulations were conducted using identical microchannel geometry, with a channel diameter of 1 mm. The physical properties of culture media and blood, specifically density and viscosity, were applied respectively, with blood as a Newtonian fluid. For the culture medium, a viscosity of 0.001 Pa·s and a density of 998 kg/m^3^ were used, while for blood, a viscosity of 0.0035 Pa·s and a density of 1,060 kg/m^3^ were assigned [78]. In the simulation setup, fluid first flows through a straight tube with a diameter of 1.5 mm. The flow then entered a steel connector tube (1.8 mm in diameter) leading into the chip. No-slip boundary conditions were applied to all channel walls. Identical inlet velocities and outlet pressures were used for both fluids to ensure a consistent basis for comparing shear stress distribution and flow profiles. The shear stress (*τ*) was calculated using the formula for Newtonian laminar flow:

$$\tau =\mu \cdot \frac{\mathrm{du}}{\mathrm{dy}}$$ (1)

where *μ* is the dynamic viscosity and $\frac{\mathrm{du}}{\mathrm{dy}}\;$ is the velocity gradient perpendicular to the wall.

To ensure the effects of fluid type, we maintained identical microchannel geometry in all stimulations, using a channel diameter of 1 mm. The physical properties of culture media and blood, such as density and viscosity, were reviewed and applied, with blood as a Newtonian fluid. In the stimulation, the fluid first flows through a straight tube with a diameter of 1.5 mm, with a consistent inlet velocity and outlet pressure to enable a direct comparison of flow characteristics. It then passed through a steel pipe (1.8 mm in diameter) that connects to the chip. No-slip boundary conditions were applied to all channel walls.

### The construction of a 3D-PVoC with the co-culture of SMCs and GFP-HUVECs

The culture medium for GFP-HUVECs is F12 and EC medium, while the culture medium for SMCs is SMCM and 1640. To prevent contamination and ensure cell growth, add 1% (v/v) penicillin–streptomycin and 10% (v/v) FBS to each culture medium, and incubate at 37 °C in 5% CO_2_ and in saturated humidity. The artificial VoC system consisted of an outer layer of GelMA encapsulating SMCs and an inner ring of GFP-HUVECs arranged in a circular pattern. Prior to cell seeding, the PDMS chip was soaked in 1% (w/v) PLL (Aladdin, Shanghai, China) for 30 min to enhance its hydrophilicity. Water contact angle measurements were then performed at specified time points (0, 24, and 48 h). Before seeding of the cells, to enhance the hydrophobic ability of the PDMS surface, the PDMS chip was soaked in 1% (w/v) PLL (Aladdin, Shanghai, China) for 1 min. Then, the steel needle with a diameter of 1 mm and a length of 100 mm was inserted into the central channel of a PDMS chip for a lumen structure of GelMA. SMCs at a cell density of 4 × 10^6^ cells ml^−1^ were dispersed in GelMA polymer solution (10%, w/v) with LAP (0.5%, w/v) and then injected into the previously processed chip model. UV light was applied rapidly for 10 s on each side, ensuring that the SMCs were evenly encapsulated within the crosslinked GelMA. After curing, the steel needle was removed, forming a hollow perfusable channel in the center, and the GFP-HUVECs medium was introduced to flush out the residual lubricant from the channel. Then, the GFP-HUVECs suspension with a density of 4 × 10^6^ cells ml^−1^ was introduced into the hollow channel and evenly spread on the inner surface of the polymerized GelMA to construct the lumen of GFP-HUVECs. For robust adhesion and growth of GFP-HUVECs and SMCs within the chip, the constructed VoC was placed in static culture for 6 h. Subsequently, a 1:1 mixture of GFP-HUVECs and SMCs media was loaded into a syringe and placed on an external pump with the flow rate set at 20 μl/h for the perfusion culture of the chip.

To observe the growth and distribution of cells in the model, the SMCs were specifically labeled with cell labeling kit Qtracker 655 (Thermo Fisher, Waltham, USA), and the 3D imaging of GFP-HUVECs and SMCs within the VoC culture system was performed using a CLSM (Zeiss LSM 900 with Airyscan2, Germany) after 6 and 24 h of culture. Maximum intensity projection images along the *z*-axis were obtained directly from the built-in image processing software, with 3 parallel samples for each time point. To assess the angiogenesis properties of GFP-HUVECs in the VoC, the Angio tool Version 0.6a (Bethesda, USA) was used for analysis, focusing on the total number of junctions, the average vessel length, vessel density, and area [79]. Data were statistically analyzed using GraphPad Prism version 8.0 (GraphPad Software, San Diego, USA).

To analyze the biological properties of the cells, VE-Cad expression of GFP-HUVECs and α-SMA expression of SMCs were investigated, respectively. In brief, the samples were fixed with 4% paraformaldehyde (PFA, Solarbio, Beijing, China) for 1 h and permeabilized with 0.1% Triton X-100 (diluted in PBS, Solarbio) for 20 min at room temperature. Add 1 ml of 5% bovine serum albumin (w/v, in PBS, Aladdin) to each sample and incubate on a shaker at room temperature for 1 h. Then, the samples were added with VE-Cad primary antibody (1:200 dilutions in PBS, Thermo Fisher), or 𝛼-SMA primary antibody (1:200 dilutions in PBS, Thermo Fisher), and incubated overnight at 4 °C, respectively. Further, Goat Anti-Rabbit IgG (H+L)-CoraLite594 for VE-Cad and Goat Anti-Rabbit IgG H&L (Alexa Fluor @ 488) for α-SMA (Thermo Fisher) were added and incubated for 2 h, respectively, and DAPI for nuclei was added for 10 min at room temperature. Notably, after each of the above reagent treatments, wash the samples 3 times with PBS for 5 min before proceeding to the next step. Finally, the images were captured using CLSM and analyzed by ImageJ software (Bethesda, USA).

### Pretreatment and morphological observation of inflammatory factor-induced M0 macrophage (MC) phenotypic transformation

To generate resting-state M0 MCs as established protocol [48], THP-1 monocytes (iCell, Shanghai, China) were seeded onto sterile coverslips and treated with 100 ng/ml phorbol 12-myristate 13-acetate (PMA, MedChemExpress, Monmouth Junction, USA) for 48 h to induce adhesion and differentiation. The medium was then replaced with fresh PMA-free THP-1 culture medium (Procell, Wuhan, China), and cells were incubated for an additional 24 h to allow complete recovery to an M0 macrophage phenotype. Subsequently, to mimic an inflammatory microenvironment, M0 MCs were stimulated for 24 h with a pro-inflammatory cocktail containing ox-LDL (50 μg/ml; Yiyuan Biotech, Guangzhou China), TNF-α (2 ng/ml; PeproTech, Cranbury, USA), and IL-1β (2 ng/ml; PeproTech) for 24 h. Untreated M0 MCs were used as the control group.

For morphological analysis, cells were subjected to cell membrane staining using fluorescently labeled wheat germ agglutinin (iF488-WGA, Servicebio, Beijing, China). Briefly, cells were washed 3 times with PBS and fixed with 4% PFA at room temperature for 15 min, and washed again 3 times with PBS (5 min each). The cells on coverslips were then incubated with 100 μl of iF488-WGA solution (5 μg/ml) at 37 °C for 30 min in the dark. Following WGA staining, nuclei were counterstained with DAPI solution for 10 min and washed 3 times with PBS for 1 min each. Coverslips were mounted using antifade mounting medium (Beyotime, Suzhou, China) and imaged using CLSM, with excitation/emission wavelengths of 488 nm (WGA) and 405 nm (DAPI). The resulting images were imported into cellpose software (developed by the MouseLand team, USA) for cell segmentation to accurately delineate individual cell boundaries. The segmented images were then processed in ImageJ using the “Analyze Particles” function to quantify morphological parameters including circularity, perimeter, and aspect ratio. Finally, the extracted data were analyzed and visualized using GraphPad Prism version 8.0.

### Recapitulation of atherogenic conditions and the development of the 3D-PAVoC

Besides the flow into the platform due to the presence of the external pumps, an inflammatory environment and hyperlipidemic factors were incorporated as AS-prone biochemical conditions. Based on our previous research, adding ox-LDL (50 μg/ml), TNF-α (2 ng/ml), and IL-1β (2 ng/ml) to the culture medium of the engineered vascular model can simulate the early pathological state of AS to some extent [24]. Similarly, to construct the 3D-PAVoC in this study, we perfused the chip with a culture medium containing the same concentrations of inflammatory factors using an external pump with a flow rate of 20 μl/h. Following this treatment for 24 h, the protein expression of VE-Cad of GFP-HUVECs in the model was observed through immunofluorescence staining and compared to the healthy control group. Z-stack overlays were generated using CLSM to obtain maximum fluorescence images. Subsequently, grayscale images were processed using ImageJ to analyze the relative cellular fluorescence area. To further understand the overall protein changes in the 3D-PVoC before and after treatment with inflammatory factors, we conducted proteomic analysis on both groups. Detailed steps can be found in the “Proteomic analysis” section. The significantly different proteins in each group were visualized using volcano plots and heatmaps.

Additionally, to verify the successful construction of the early-stage AS disease model, TG, CH, ROS, and ox-LDL secretion were measured in various groups based on the manufacturer’s instructions for ELISA kits (Mskbio, Austin, USA). Notably, the collected medium from the perfusion culture was mixed with the co-cultured cell samples from the chip. The mixture was then processed with an ultrasonic disruptor (Scientz, Ningbo, China) for 1 min (ultrasonication for 5 s at 200 W, interval 5 s), followed by centrifugation at 2,000 rpm for 5 min, and the supernatant was collected for ELISA detection.

### RAP evaluation

After inflammatory and hyperlipidemic stimulation, the RAP (Aladdin) was selected as a model drug to investigate the cell behaviors at various RAP concentrations from 0 to 100 μM in the group of the 3D-PAVoC, along with the group of the cells in the 2D plate and the 3D-PVoC as control. Cell viability and proliferation with drug treatment were assessed using the CCK-8 assay (Solarbio). Briefly, for the 2D plate, a mixture of 2,500 GFP-HUVECs and 2,500 SMCs per well was seeded into a 96-well microplate and incubated for 24 h. Then, discard the medium and pipette RAP (100 μl) concentrations from 0 to 100 μM with each concentration in 6 replicates to the 96-well plate and culture for 24 h, along with the group without RAP treatment as the negative control. After washing twice with PBS, 10 μl of CCK-8 working solution was added to each well, followed by 100 μl of fresh culture medium, and incubated for 3 h. Finally, measure the optical densities (OD) at 450 nm of each well using a microplate reader (Thermo Fisher). Based on the measured OD values, the dose–response curve for RAP concentrations ranging from 0 to 100 μM was fitted using the GraphPad Prism 8.0 software, and the IC_50_ values for each group were calculated accordingly. Notably, after the perfusion culture, samples from the 3D-PVoC and 3D-PAVoC were transferred into a 96-well plate and treated with CCK-8 working solution.

### Proteomic analysis

The cells were collected from the groups of 2D plate, 3D-PVoC, 3D-PAVoC, and 3D-PAVoC drug treatment using the concentration optimized from the “The construction of a 3D-PVoC with the co-culture of SMCs and GFP-HUVECs” section. Each group had 3 parallel samples. The 2D plate was directly digested with trypsin, followed by centrifugation and supernatant removal twice. For all the 3D groups, the samples were treated with GelMA lysis buffer (EFL) for 30 min to fully lyse the hydrogel blocks and retrieve the cells after centrifuging at 100 rpm for 5 min. The supernatant was discarded, and the pellet was washed with 5 ml of PBS and centrifuged again. The resulting cell pellet was rapidly frozen in liquid nitrogen and then stored at −80 °C until needed.

Using TMT labeling technology for detection, protein samples from each group were first subjected to enzymatic digestion and desalting. A strong reducing agent, dithiothreitol (Amresco, Cleveland, USA), was added to break disulfide bonds, followed by the addition of iodoacetamide (Amresco) to alkylate the thiol groups. Then, trypsin was added to digest the proteins, and the samples were incubated overnight at 37 °C. The next day, formic acid (Sigma-Aldrich, St. Louis, USA) was added to adjust the pH to below 3 to stop the enzymatic digestion. Finally, a C18 desalting column (Waters Corporation, Milford, USA) was used to remove salts and other impurities from the samples. Next, TMT16plex Isobaric Label Reagent Set (Thermo Fisher) was added to the digested samples and allowed to react at room temperature for 1 h. Ammonia (Wako Pure Chemical Industries Ltd, Chuo-ku, Japan) was added to stop the reaction. After that, the samples were fractionated. The mixed labeled samples were dissolved in 100 μl of mobile phase A and centrifuged at 14,000 *g* for 20 min, and the supernatant was collected. The samples were then fractionated using high-performance liquid chromatography (Purkinje General Electric Technology Co., Ltd., RIGOL L-3000, Beijing, China). Subsequently, liquid chromatography–tandem mass spectrometry was performed for mass spectrometry analysis. The mass spectrometry data were analyzed for protein identification, quantification, and interaction using the *Homo sapiens* database in UniProtKB. These data were then converted and compared with the GO and KEGG databases for gene function annotation and functional enrichment analysis, as well as for metabolic pathway and signal transduction pathway analysis.

### mRNA sequencing

The preparation of samples was operated as described in the “Proteomic analysis” section. The samples were centrifuged to remove the supernatant, leaving behind cell clusters. These cell clusters were subsequently lysed using Trizol reagent (Invitrogen) and stored at −80 °C. mRNA sequencing was performed using the Illumina platform, followed by bioinformatics analysis on the Cloud Platform provided by Qingke Biotechnology (Guangzhou, China).

### Mouse model of AS

All the experimental procedures utilizing animals were conducted in accordance with protocols approved by the Tenth of Affiliated Hospital, Southern Medical University. To establish a reproducible mouse model of AS with plaque formation, male ApoE^−/−^ mice on a C57BL/6 genetic background (6 weeks old) were maintained under standard laboratory conditions (temperature: 20 to 26 °C and humidity: 40% to 70%) and fed a high-fat diet (SBF, SFD009, China) for 14 weeks, until they reached 20 weeks of age. The development of atherosclerotic lesions was confirmed through en face ORO staining of the whole aorta, histological analysis of aortic cross-sections using H&E staining, and serum lipid profiling.

### In vivo anti-atherosclerotic evaluation of RAP

After 20 weeks of high-fat diet feeding, ApoE^−/−^ mice were randomly assigned to 2 groups (*n* = 6 per group) and received daily intraperitoneal injections of either physiological saline (500 μl) or RAP (55.36 μM in 500 μl) for 4 weeks. RAP was first dissolved in dimethyl sulfoxide (DMSO, Solarbio, Beijing, China) at a stock concentration of 10 mM, then diluted in sterile physiological saline to a final injection concentration of 55.36 μM, resulting in a final DMSO content of 0.55%. To control for any potential effects of the vehicle, the saline control group received an equivalent volume (500 μl) of saline containing 0.55% DMSO. Prior to sacrifice, in vivo ultrasound imaging of the aortic root was performed for each group using the Vevo 3100 high-resolution imaging system (FUJIFILM VisualSonics Inc., Toronto, Canada) equipped with an MX400 linear-array transducer (30 MHz). Mice were anesthetized with 1% to 2% isoflurane (RWD Life Science Co., Ltd., Shenzhen, China) and positioned supine on a temperature-controlled platform with integrated ECG and respiratory monitoring. B-mode images were acquired from the parasternal long-axis view, and image analysis was performed using ImageJ software. At the end of treatment period, blood was collected via retro-orbital bleeding. Plasma was isolated by centrifugation and analyzed using commercial assay kits (Rayto, Shenzhen, China) to measure levels of CH, TG, LDL, and HDL. Following blood collection, mice were euthanized, and the entire aorta (from heart to iliac bifurcation) was dissected, fixed in 4% PFA for 24 h, and carefully cleared of perivascular fat. The aorta was then longitudinally opened, stained with ORO (Servicebio, Wuhan, China), and imaged to evaluate en face plaque area. For histological evaluation, the aortic sinus was harvested, cryosectioned, and stained with ORO and hematoxylin. Slides were scanned using the PANNORAMIC MIDI digital slide scanner (Pannoramic MlDl, 3DHISTECH, Hungary) and analyzed with ImageJ software. Plaque burden was quantified as the percentage of ORO-positive area relative to the total aortic or aortic root area.

### Histology and immunohistochemistry staining of the aortic sinus

Histological analysis of the aortic sinus was performed in ApoE^−/−^ mice following daily intraperitoneal injections of either physiological saline (500 μl) or RAP (55.36 μM in 500 μl) for 4 weeks. Serial frozen sections were prepared from each sample for various staining procedures. H&E staining was used to quantify necrotic core area, while Masson’s trichrome staining (Servicebio) was employed to assess collagen content within the lesions. Macrophage infiltration was evaluated by immunohistochemistry using a primary CD68 antibody (1:500 dilution, GB113109, Servicebio) and horseradish peroxidase-labeled goat anti-rabbit secondary antibody (1:200 dilution, GB23303, Servicebio), and stained slides were scanned using the PANNORAMIC MIDI system. Quantification of histological features was conducted using ImageJ software. To assess potential systemic toxicity of RAP treatment, major organs including the heart, liver, spleen, lungs, and kidneys were collected, paraffin-embedded, sectioned, and stained with H&E for histopathological examination.

### Statistical analysis

Statistical analysis was performed using GraphPad Prism version 8.0. Data are presented as mean ± SD, and statistical significance was defined as a *P* value < 0.05. Comparisons between the 2 groups were made using an unpaired Student’s *t* test. For comparisons among multiple groups, one-way analysis of variance (ANOVA) was used. All experiments were conducted with at least 3 replicates, and data were checked for normal distribution and homogeneity of variances before analysis.

## Data Availability

Data will be made available on request.
