# Supplementary material for: Microfluidic Perfusable Pathological Vasculature for Atherosclerosis Drug Screening
Source: Research (Wash D C). 2025 Sep 19;8:0902. doi: 10.34133/research.0902 (PMC12446761; doi:10.34133/research.0902)
Supplement: Supplementary 1 — Figs. S1 to S8 Tables S1 and S2 [file research.0902.f1.docx]

*Supporting information*

**Microfluidic Perfusable Pathological Vasculature for Atherosclerosis Drug Screening**

Jing Liu^1,2^, Mulan Zhu^1,2^, Na Bai^1,2^, Nan Huang^2,3^, Wentai Zhang^1,2^, Zhilu Yang^1,2,^*, Ying Wang^1,2,^*

^1^The Tenth Affiliated Hospital, Southern Medical University, Dongguan, Guangdong, 523059, China. ^2^Shenzhen Clinical Medical School, Southern Medical University, Guangzhou, Guangdong, 510515, China. ^3^GuangZhou Nanchuang Mount Everest Company for Medical Science and Technology, Guangzhou, 510670, China

*Address correspondence to: zhiluyang1029@smu.edu.cn (Z.Y.); wangying277@outlook.com (Y.W.)


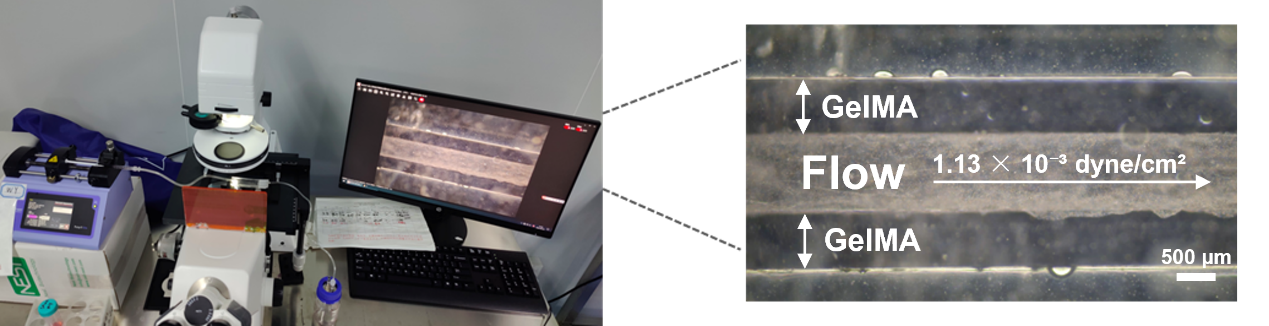


**Fig. S1.** Photograph of fluid flow inside a microfluidic VOC.

*
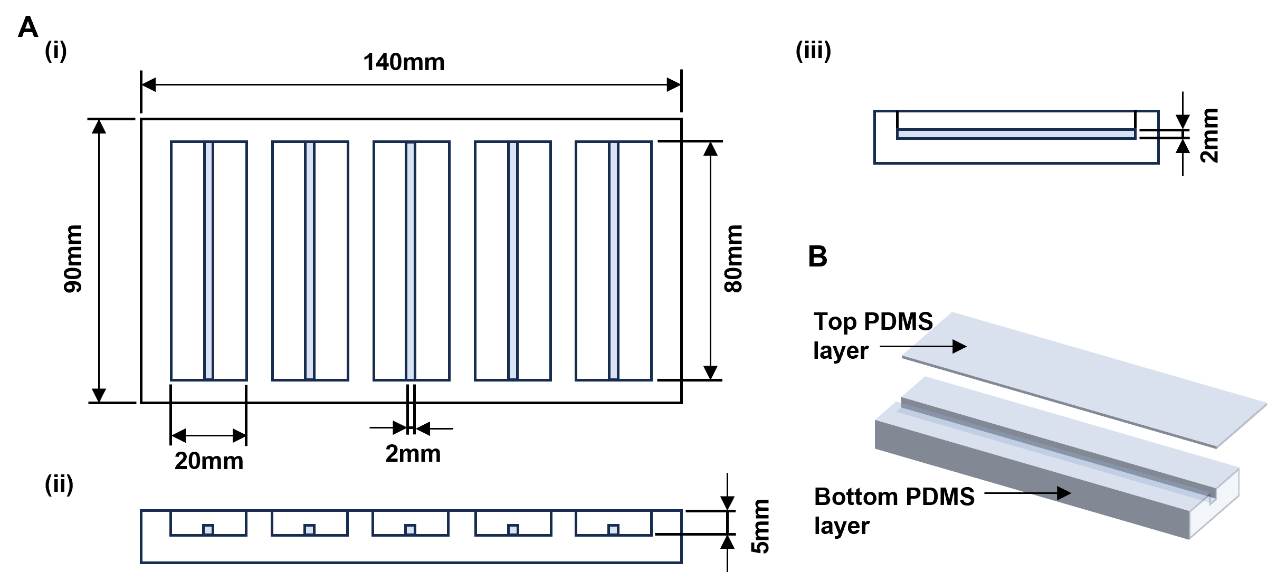
*

**Fig. S2.** The CAD design of the PDMS chip. (A) Top view(i), front view(ii), and side view (iii) of the PMMA mold. (B) Schematic diagram of the microfluidic VOC.

*
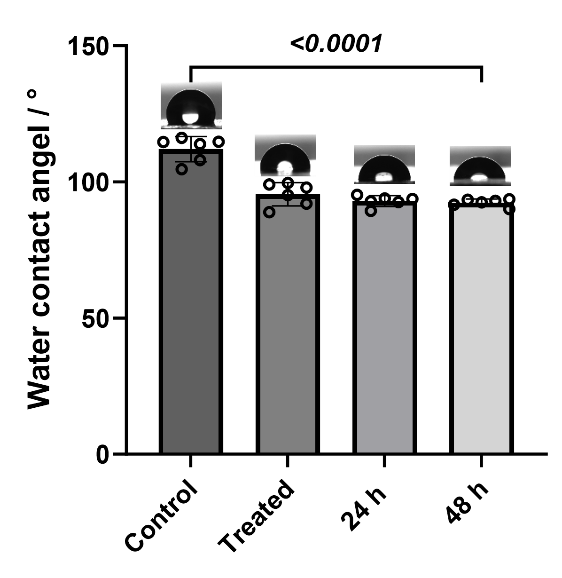
*

**Fig. S3.** Water contact angle measurements of hydrogel surfaces before and after PLL treatment at various time points. Four groups were analyzed: untreated control, immediately after PLL treatment (0 h), and at 24 h and 48 h post-treatment. The results showing the dynamic changes in surface hydrophilicity over time following PLL modification. Data are presented as mean ± SD (n = 6). Statistical significance was assessed using one-way ANOVA followed by Tukey’s post hoc test; p < 0.05 was considered significant.

*
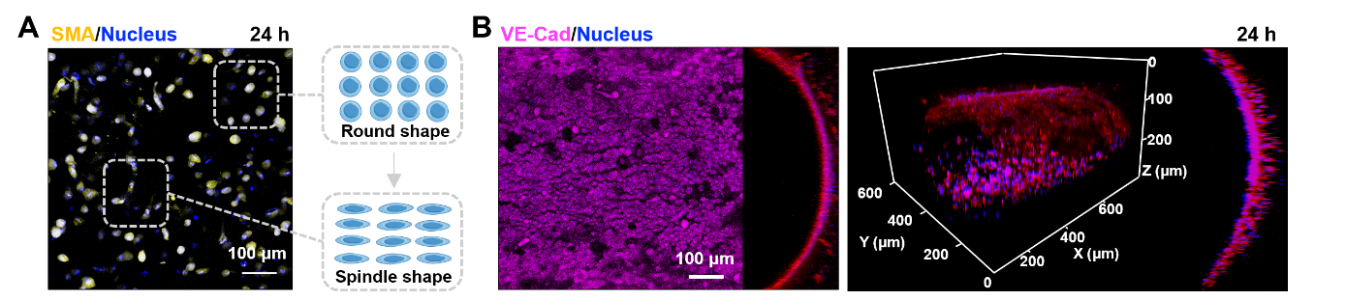
*

**Fig. S4.** (A) CLSM images of α-SMA in SMCs and VE-cad expression in GFP-HUVECs after 24 h perfusion culture, and (B) shows that VE-cad is ringed in the model.

*
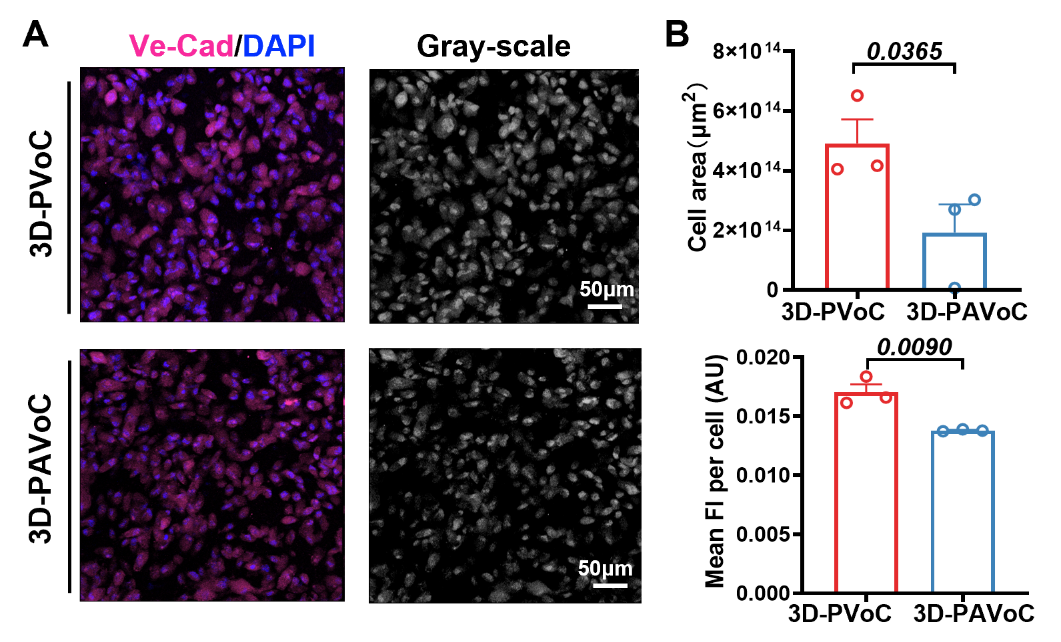
*

**Fig. S5.** (A) CLSM images showing VE-cad expression in the 3D-PAVoC group are compared with those without treatment with inflammatory cytokines. (B) Statistical analysis of cell area and average fluorescence intensity performed using gray-scale images.

**
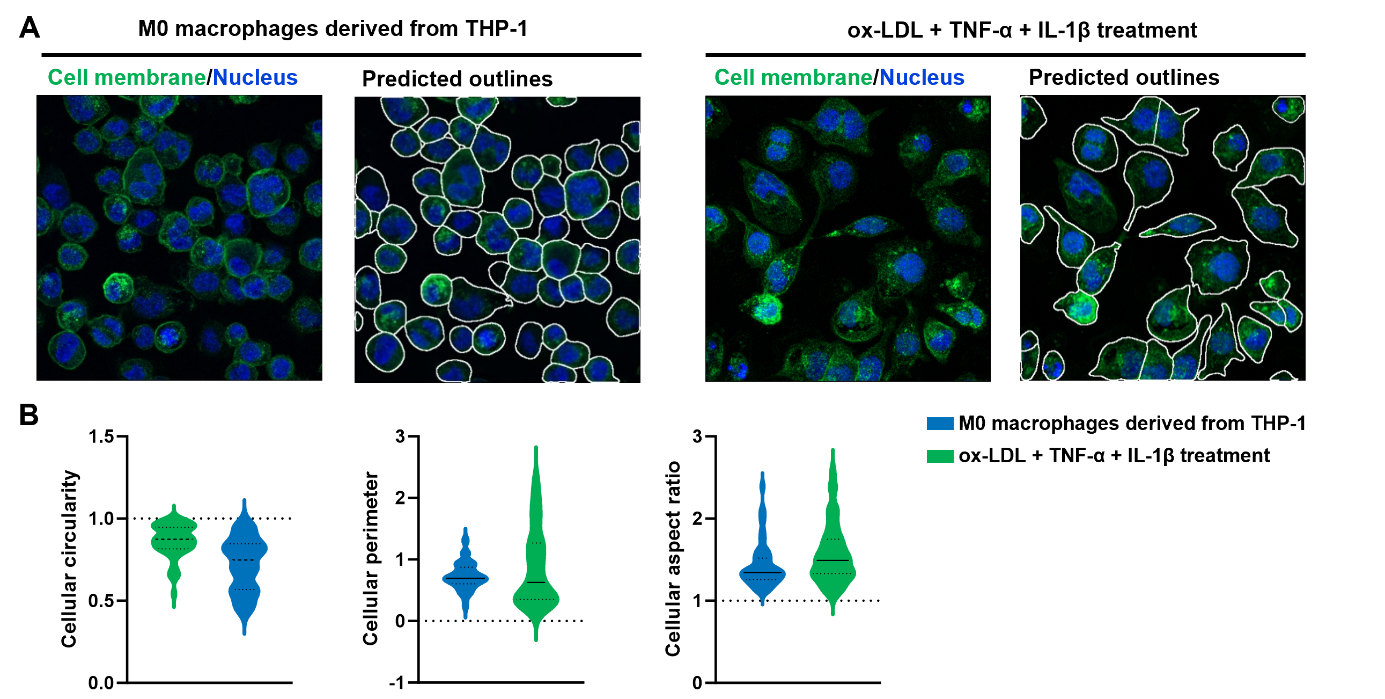
**

**Fig. S6.** Morphological changes in macrophages (MCs) induced by ox-LDL and inflammatory stimuli. (A) The fluorescence images and predicted cellular outlines of THP-1-derived M0-type MCs and MCs treated with ox-LDL, TNF-α, and IL-1β stained with WGA (green) and DAPI (blue). (B) Quantification of morphological parameters, including cellular circularity, perimeter, and aspect ratio. Data are displayed as violin plots, with statistical comparisons between treated and untreated groups performed using a two-tailed independent-samples t-test. ns indicates no statistically significant difference.

***
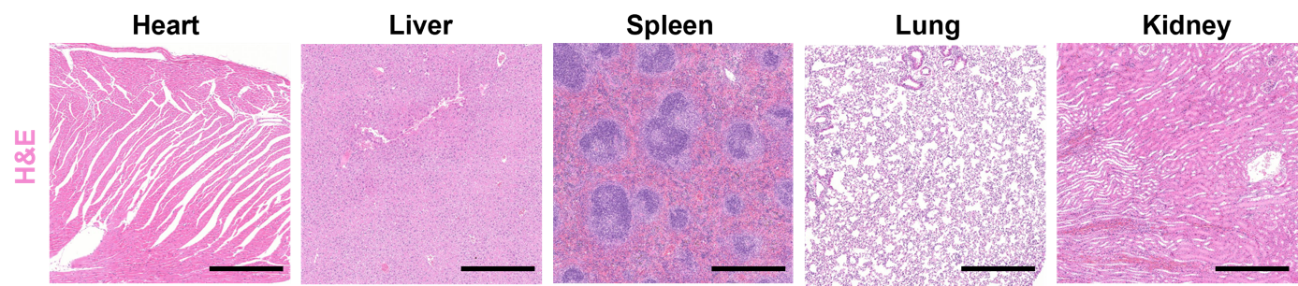
***

**Fig. S7.** Histopathological evaluation of RAP toxicity in major organs. Representative H&E-stained sections of the heart, liver, spleen, lung, and kidney were collected from ApoE⁻/⁻ mice after 4 weeks of RAP treatment. No overt histological abnormalities were observed in any of the examined organs, indicating that the effective concentration of RAP used in this study does not cause detectable systemic toxicity. Scale bar = 500 μm.

*
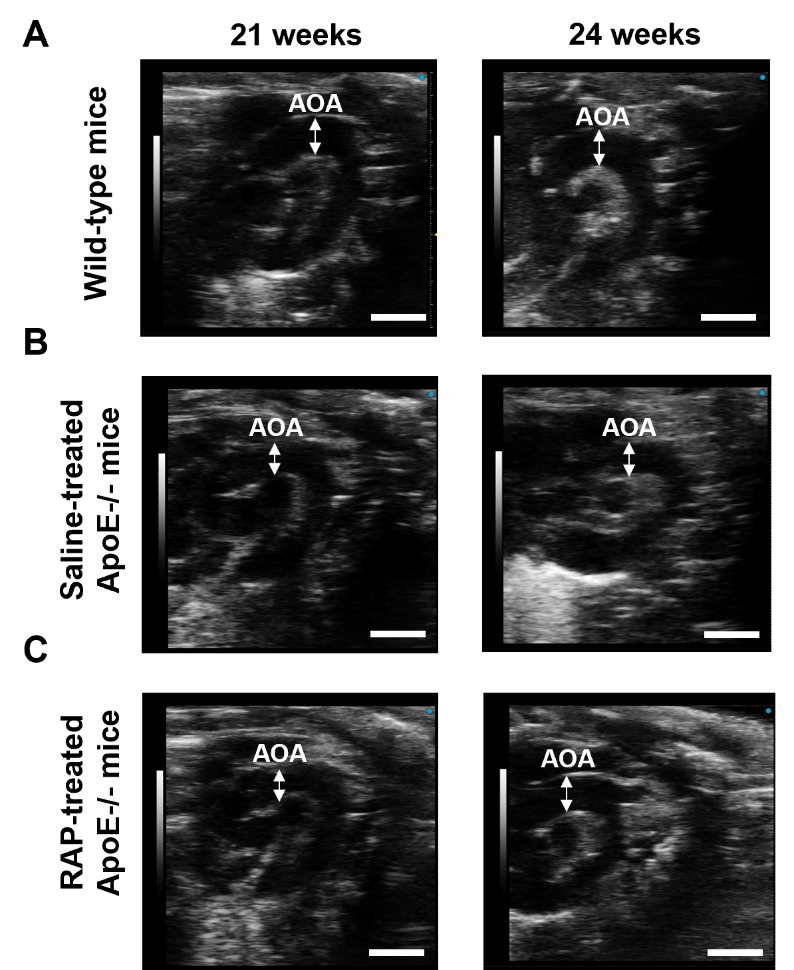
*

**Fig. S8.** Longitudinal assessment of aortic root morphology via ultrasound in wild-type mice and ApoE⁻/⁻ mice under RAP or saline treatment. (A) Representative B-mode ultrasound images of the aortic root in a healthy C57BL/6 mouse at weeks of 21 and 24, illustrating physiological vascular morphology. (B) ApoE^-/-^ mice fed a high-fat diet (HFD) until 20 weeks of age and subsequently administered saline. The aortic ultrasound images were obtained at 1- and 4-weeks post-treatment to assess lesion progression without pharmacological intervention. (C) ApoE^-/-^ mice under the same HFD regimen were treated with RAP starting at the week of 20. The ultrasound images were collected at 1- and 4-weeks post-treatment to evaluate therapeutic effects. Scale bar=2 mm.

**Table S1.** KEGG pathway analysis and comparison (RAP (55.36Μm) vs 3D-PAVoC)

| **KEGG pathway ID** | **KEGG pathway description** | **Up Gene ID** | **Down Gene ID** |
| --- | --- | --- | --- |
| ko04060 | **Cytokine-cytokine receptor interaction** | IL1RN/NGF/GDF15/GDF9/ACKR3/IFNLR1/BMP8B/NGFR/CCL3/IL24/CCL5/BMP6/GDF1/IL36B/CCL7/CXCL8/CSF3/IL13RA2/CXCL5/GDF7/OSM/CSF2/IL6/IL1B | TNFRSF19/TNFSF15/TNFSF18/TNFSF10/TNFSF4/IL21R/IL31RA/IFNE/TNFRSF9/IL20RA/LTB/CLCF1/IL20RB/CXCR4/TNFRSF6B/TGFBR2/IL18/IL6R/TNFRSF10C |
| ko04668 | **TNF signaling pathway** | VEGFC/IL1B/CREB3L1/TNF/IL6/CSF2/TRAF1/MMP3 | gene:ENSG00000289720/BIRC3/VCAM1/CCL5/NOD2/CASP10/JAG1/CFLAR/PIK3R3/FAS/BCL3 |
| ko05200 | Pathways in cancer | VEGFC/PDGFRB/MMP1/GSTM1/CXCL8/LAMA4/IL6/FRAT1/GSTM5/FGF2/STAT5A/HMOX1/BDKRB2/WNT5A/CXCL12/RUNX1T1/HSP90AA1/LAMC3/IL12A/TRAF1/PIM1/RET/MMP2/IL7R/HEYL | CXCR4/SHH/gene:ENSG00000287856/E2F2/MDM2/TGFA/SLC2A1/EDNRB/EGLN1/FGF18/PTCH2/RASSF5/WNT4/COL4A6/CCNE2/BIRC3/FZD10/CDH1/CDKN2A/IL6R/VEGFA/GNG3/JAG1/CCND3/ESR2/WNT2B/PIK3R3/FAS/PDGFB |
| ko04061 | Viral protein interaction with cytokine and cytokine receptor | TNF/IL24/IL6/CXCL12/ACKR3/CXCL8 | CXCR4/IL20RB/IL18/CCL5/CCL3L1/CCL3/IL6R/CCL8/TNFSF14/TNFRSF10D/CCR7 |
| ko05418 | **Fluid shear stress and atherosclerosis** | IL1B/KLF2/GSTM1/TNF/GSTM5/CTSL/HMOX1/ARHGEF2/HSP90AA1/SQSTM1/MMP2 | VCAM1/SDC4/MEF2C/VEGFA/PLAT/CAV1/PIK3R3/PECAM1/PDGFB |
| ko04514 | Cell adhesion molecules | CD274/ITGA4/PDCD1LG2/HLA-DPB1/NFASC/CLDN23/NLGN4Y/JAM3 | NTNG1/NLGN3/CLDN4/VCAM1/SDC4/CDH1/CDH3/NRCAM/ITGB8/CLDN7/VCAN/PECAM1 |
| ko05134 | Legionellosis | TNF/HSPA1A/IL1B/IL6/HSPA8/IL12A/HSPA1L/HSPA1B/CXCL8 | IL18/BNIP3 |
| ko05219 | Bladder cancer | MMP1/MMP2/HBEGF/CXCL8 | CDKN2A/E2F2/VEGFA/MDM2/CDH1 |
| ko04510 | Focal adhesion | COL1A2/VEGFC/FLNC/ITGA4/PDGFRB/LAMA4/ITGA11/MYL5/COL1A1/LAMC3 | COL9A3/COL4A6/ITGB6/BIRC3/VAV3/ITGA10/VEGFA/CAV1/CCND3/ITGB8/PIK3R3/SHC4/PDGFB/COL9A2 |
| ko04933 | AGE-RAGE signaling pathway in diabetic complications | TNF/STAT5A/COL1A2/VEGFC/IL6/IL1B/PIM1/COL1A1/MMP2/EGR1/CXCL8 | PIK3R3/COL4A6/VEGFA/VCAM1 |
| ko04066 | **HIF-1 signaling pathway** | HMOX1/IL6/ANGPT1 | EGLN1/ENO1/LDHA/PIK3R3/ALDOA/ALDOC/IL6R/GAPDH/gene:ENSG00000287856/VEGFA/PGK1/PDK1/SLC2A1 |
| ko01230 | Biosynthesis of amino acids | ABHD14A-ACY1/ASNS | ENO1/PGAM1/ALDOA/ALDOC/GAPDH/PGK1/PGAM4/LMNB1/TPI1/PC |
| ko04512 | ECM-receptor interaction | COL1A2/ITGA11/LAMA4/ITGA4/COL1A1/LAMC3 | ITGA10/COL4A6/ITGB6/SDC4/COL9A3/ITGB8/COL9A2 |
| ko04360 | Axon guidance | NRP1/MYL5/WNT5A/CXCL12/UNC5B | CXCR4/SHH/NTNG1/EFNA3/EFNA1/WNT4/EPHA3/SEMA6A/PDK1/SEMA3C/ROBO2/NFATC2/SEMA4A/UNC5A/PIK3R3/SEMA4B |
| ko04670 | Leukocyte transendothelial migration | MYL5/CXCL12/CLDN23/ITGA4/JAM3/MMP2 | CXCR4/CLDN7/RAPGEF4/RASSF5/VAV3/PIK3R3/PECAM1/CLDN4/VCAM1 |
| ko05142 | Chagas disease | TNF/BDKRB2/IL6/IL1B/IL12A/CXCL8 | GNAL/PIK3R3/CCL3L1/CCL3/FAS/GNA15/CCL5/CFLAR |
| ko05205 | Proteoglycans in cancer | FLNC/TNF/CTSL/HBEGF/HSPB2/FGF2/WNT5A/DCN/MMP2 | SHH/MDM2/WNT4/FZD10/SDC4/PTPN6/VAV3/VEGFA/CAV1/WNT2B/PIK3R3/FAS/ANK3 |
| ko04610 | Complement and coagulation cascades | C5/F2RL2/BDKRB2/SERPING1 | SERPINA1/CFD/gene:ENSG00000273259/PLAT/MASP2/C5AR1/gene:ENSG00000284969/CFB |
| ko00010 | **Glycolysis /Gluconeogenesis** |  | ENO1/GPI/LDHA/PGAM1/ALDOA/GAPDH/PGK1/PGAM4/CCND3/TPI1/ALDOC |

**Table S2.** KEGG pathway analysis and comparison (RAP (55.36Μm) vs 3D-PVoC)

| **KEGG pathway ID** | **KEGG pathway description** | **Up Gene ID** | **Down Gene ID** |
| --- | --- | --- | --- |
| ko04060 | **Cytokine-cytokine receptor interaction** | IL1RN/NGF/GDF15/GDF9/ACKR3/IFNLR1/BMP8B/NGFR/CCL3/IL24/CCL5/BMP6/GDF1/IL36B/CCL7/CXCL8/CSF3/IL13RA2/CXCL5/GDF7/OSM/CSF2/IL6/IL1B | TNFRSF19/TNFSF15/TNFSF18/TNFSF10/TNFSF4/IL21R/IL31RA/IFNE/TNFRSF9/IL20RA/LTB/CLCF1/IL20RB/CXCR4/TNFRSF6B/TGFBR2/IL18/IL6R/TNFRSF10C |
| ko03030 | DNA replication | \ | PCNA/MCM4/RFC5/RFC3/LIG1/FEN1/POLE2/RFC2/MCM3/MCM5/POLE/POLA2 |
| ko01523 | Antifolate resistance | IL6/ALOX12/ABCC3/IL1B | FOLR1/ABCC2/NFKB1/TYMS/DHFR/ABCG2 |
| ko04657 | IL-17 signaling pathway | CSF3/CXCL5/IL6/MMP3/LCN2/CCL7/JUN/CSF2/PTGS2/MMP1/IL1B/CXCL8 | NFKB1/S100A9/SRSF1/MUC5AC/IKBKE |
| ko04360 | Axon guidance | ROBO3/EFNA4/SEMA4G/PIK3CD/WNT5A/PLCG2/ABLIM2/RGS3/PTCH1/GDF7/RND1 | EPHA3/UNC5A/EFNB2/SEMA3A/CXCR4/PDK1/ABLIM1/EPHA1/SEMA3C/EPHA4/EFNA1/SEMA3B/PIK3R3/SHH/NFATC2 |
| ko04668 | **TNF signaling pathway** | IL6/MMP3/PIK3CD/CCL5/JUN/CSF2/PTGS2/CXCL5/IL1B | CFLAR/MAP2K3/BIRC3/JAG1/CASP10/NFKB1/gene:ENSG00000289720/MAP2K6/PIK3R3 |
| ko04110 | Cell cycle | CDKN1C | CDC45/CDKN2C/CCNE2/PCNA/MDM2/CDC25A/CCND3/MCM3/PKMYT1/E2F1/RBL1/CDC6/SMC1A/MCM4/MCM5/E2F2/WEE1/TFDP1 |
| ko05142 | Chagas disease | PIK3CD/CCL3/CCL5/JUN/IL1B/IL6/CXCL8 | ADCY1/TLR6/CFLAR/GNA15/TGFBR2/NFKB1/PIK3R3/TLR4/GNAL |
| ko05200 | Pathways in cancer | FGF2/COL4A3/PLD1/MMP2/WNT11/DLL1/PIK3CD/PLCG2/PTGS2/WNT5A/MMP1/CXCL8/RASGRP1/PRKCG/HMOX1/PTCH1/LAMC3/RUNX1T1/IL6/JUN | ADCY1/CCNE2/GSTT2B/MDM2/GNG4/CCND3/KITLG/PDGFB/BIRC3/GNG2/LPAR6/E2F1/NFKB1/CDH1/CXCR4/RASSF5/ZBTB16/PTGER2/FGF18/BIRC5/EGLN3/PIK3R3/RAD51/RARB/TGFA/JAG1/FZD10/COL4A6/TGFBR2/E2F2/IL6R/SHH/ITGA2/HGF |
| ko04218 | Cellular senescence | PIK3CD/CXCL8/SQSTM1/IL6 | MAP2K3/CCNE2/MYBL2/MDM2/IGFBP3/CCND3/CDC25A/E2F1/RBL1/NFKB1/FOXM1/RASSF5/MAP2K6/PIK3R3/TGFBR2/E2F2/NFATC2 |
| ko01521 | EGFR tyrosine kinase inhibitor resistance | IL6/FGF2/PIK3CD/NRG1/PLCG2/PRKCG/SHC2 | PDGFB/SHC4/IL6R/TGFA/PIK3R3/HGF |
| ko04061 | Viral protein interaction with cytokine and cytokine receptor | IL6/ACKR3/CCL3/IL24/CCL5/CXCL5/CCL7/CXCL8 | IL20RA/IL20RB/TNFSF10/IL18/IL6R/CXCR4/TNFRSF10C |
| ko05134 | Legionellosis | IL6/HSPA1B/HSPA1A/HSPA1L/IL1B/HSPA8/CXCL8 | NFKB1/IL18/TLR4 |
| ko01110 | Biosynthesis of secondary metabolites | FLCN/gene:ENSG00000264187 | \ |
| ko00670 | One carbon pool by folate | gene:ENSG00000283189/AMT | KRT81/KRT86/TYMS/DHFR |
| ko03430 | Mismatch repair |  | EXO1/PCNA/RFC5/RFC3/LIG1/RFC2 |
| ko04620 | Toll-like receptor signaling pathway | IL6/PIK3CD/CCL3/CCL5/JUN/IL1B/CXCL8 | TLR6/MAP2K3/NFKB1/MAP2K6/PIK3R3/SPP1/IKBKE/TLR4 |
| ko05206 | MicroRNAs in cancer | BMF/PLCG2/PTGS2/TNXB/PRKCG/HMOX1 | CCNE2/MDM2/CDC25A/PDGFB/E2F1/NFKB1/KIF23/SERPINB5/DNMT1/ST14/E2F2/CDCA5/TP63/SHC4 |
| ko04933 | AGE-RAGE signaling pathway in diabetic complications | IL6/PIK3CD/JUN/IL1B/PLCG2/EGR1/COL4A3/MMP2/CXCL8 | F3/COL4A6/TGFBR2/NFKB1/PIK3R3 |
